# Antihypertensive drugs for hyperuricemia in patients with hypertension: a systematic review and network meta-analysis of Chinese trials

**DOI:** 10.1186/s12872-025-05339-7

**Published:** 2025-12-02

**Authors:** Juan Wu, Hong Di, Yun Zhang, Xuejun Zeng

**Affiliations:** Department of General internal medicine, Peking Union Medical College Hospital, Chinese Academy of Medical Science & Peking Union Medical College, No. 1 Shuaifuyuan, Dongcheng District, Beijing, 100730 P.R. China

**Keywords:** Antihypertensive drugs, Hyperuricemia, Systematic review, Network meta-analysis

## Abstract

**Objective:**

This study aimed to compare and rank antihypertensive agents based on their urate-lowering effects in patients with hypertension and hyperuricemia.

**Methods:**

We systematically searched PubMed, Embase, the Cochrane Library, the Chinese National Knowledge Infrastructure, and the Wanfang databases for eligible trials published up to September 2023. Randomized controlled trials that directly compared different antihypertensive agents in hypertensive patients with hyperuricemia were included. Pairwise and network meta-analyses were conducted using odds ratios and weighted mean differences with 95% confidence intervals. The surface under the cumulative ranking area (SUCRA) was used to rank the efficacy of the antihypertensive treatments.

**Results:**

A total of 172 trials involving 16,226 hypertensive patients with hyperuricemia were included in the final analysis. According to SUCRA values, losartan plus amlodipine (SUCRA: 96%), valsartan plus amlodipine (SUCRA: 90%), and allisartan (SUCRA: 90%) showed relatively superior effects in reducing serum uric acid levels. Additionally, irbesartan plus amlodipine (SUCRA: 94%), losartan plus amlodipine (SUCRA: 92%), and losartan (SUCRA: 84%) were associated with higher effective rates.

**Conclusion:**

Based on the only available eligible evidence, the combination of losartan and amlodipine was the optimal dual therapy for Chinese patients with hypertension and hyperuricemia, while allisartan and losartan were the most effective monotherapies for lowering serum uric acid. These findings are not generalizable to non-Chinese populations, reflecting a gap in global evidence rather than intentional study scope restriction.

**Supplementary Information:**

The online version contains supplementary material available at 10.1186/s12872-025-05339-7.

## Introduction

According to the 2015 China Health and Nutrition Survey, the prevalence of hypertension in China was 27.9%, compared to a global prevalence of 31.1% among adults. Substantial and growing disparities in hypertension rates have been observed across different countries [1, 2]. Hypertension is commonly associated with comorbidities such as hyperuricemia and gout. The prevalence of hyperuricemia among hypertensive patients in central and east European countries is approximately 25% [3, 4], whereas in China, this figure reaches as high as 38.7% [5]. Moreover, data from the National Health and Nutrition Examination Survey indicate that nearly 74% of gout patients also have hypertension [6]. Studies have shown that hypertension accompanied by hyperuricemia or gout significantly increases the risk of cardiovascular disease, renal insufficiency, and all-cause mortality [7, 8]. Therefore, identifying effective treatments to prevent and mitigate of hyperuricemia is of great importance.

The potential influence of antihypertensive agents on hyperuricemia has long been a topic of clinical interest. However, findings have been inconsistent due to a limited number of studies on this subject. Some evidence suggests that the use of diuretics, β-blockers, and α-blockers may increase the risk of hyperuricemia [9], while calcium channel blockers, angiotensin-converting enzyme inhibitors, and angiotensin II receptor blockers appear to have no significant effect [10]. In contrast, a nationwide study in China reported that the use of losartan, valsartan, nifedipine, and β-blockers was associated with a significant reduction in hyperuricemia risk [5]. Despite these findings, most existing research has focused on the association between antihypertensive medications and hyperuricemia risk. It remains unclear whether antihypertensive agents can improve outcomes in hypertensive patients who already have hyperuricemia.

A previous systematic review included 31 randomized controlled trials (RCTs) to evaluate the effects of losartan in hypertensive patients with hyperuricemia. It concluded that losartan is more effective than other antihypertensive agents in reducing serum uric acid (SUA) levels and should be recommended for this patient population [11]. Nevertheless, no study has systematically compared or ranked the therapeutic effects of different antihypertensive agents on hyperuricemia using direct and indirect evidence. Given the ongoing controversy regarding optimal treatment strategies for hypertensive patients with hyperuricemia—across all populations—we designed this systematic review and network meta-analysis to synthesize global evidence on the urate-lowering efficacy of antihypertensive agents. Our primary goal was to provide broadly applicable evidence-based guidance for clinical practice worldwide. However, during the literature screening process, we identified a critical gap in the global evidence base: no non-Chinese RCTs met our full inclusion criteria. This unplanned geographic concentration of data reflects a lack of relevant research in non-Chinese populations, not a deliberate exclusion of non-Chinese studies. We therefore proceeded with synthesizing the available Chinese data to address the unmet need for evidence in this patient group, while explicitly acknowledging that the resulting findings are constrained by the limited global evidence.

## Methods

### Search strategy and selection criteria

This systematic review and network meta-analysis were conducted and reported in accordance with the Preferred Reporting Items for Systematic Reviews and Meta-Analyses (PRISMA) guidelines [12]. The study protocol was registered on the INPLASY platform (registration number: INPLASY2023100059). Eligible studies included RCTs that directly compared different antihypertensive agents in hypertensive patients with hyperuricemia. No restrictions were applied regarding publication language or geographic origin of studies. We systematically searched PubMed, Embase, the Cochrane Library, the Chinese National Knowledge Infrastructure (CNKI), and the Wanfang database for relevant articles published up to September 2023. To optimize search sensitivity, a combination of controlled vocabulary (MeSH terms for PubMed and the Cochrane Library, Emtree for Embase) and free-text keywords was used for each database. The search strategy was further supplemented with specific drug names and combination regimens to ensure comprehensive retrieval of studies on particular antihypertensive treatments. These included monotherapies (allisartan, amlodipine, benazepril, candesartan, captopril, enalapril, felodipine, fosinopril, irbesartan, lisinopril, losartan, nifedipine, perindopril, telmisartan, valsartan) and combination therapies (e.g., irbesartan plus amlodipine, irbesartan plus hydrochlorothiazide [HCTZ], irbesartan plus nifedipine, losartan plus amlodipine, losartan plus nifedipine, valsartan plus amlodipine, valsartan plus nifedipine). These terms were combined with controlled vocabulary and free-text keywords related to hyperuricemia and RCTs. The complete search strategies for each database are available in Supplemental File 1. Additionally, we searched the ClinicalTrials.gov website (US NIH) for unpublished completed trials and manually examined the reference lists of relevant review articles to identify other potentially eligible studies.

Two authors (HX and DH) independently performed the literature search and study selection. Any disagreements were resolved through group discussion until consensus was reached. Studies were included if they met the following criteria: (1) Participants were aged ≥ 18 years and diagnosed with both hypertension and hyperuricemia. Hypertension was defined according to the 2018 Chinese Guidelines for the Management of Hypertension (systolic blood pressure ≥ 140 mmHg and/or diastolic blood pressure ≥ 90 mmHg on two separate measurements, or current use of antihypertensive medication for previously diagnosed hypertension). Hyperuricemia was defined based on the 2020 Chinese Guidelines for the Management of Hyperuricemia and Gout. Minor variations in diagnostic thresholds across trials are documented in Supplemental File 2, though all included studies adhered to the core diagnostic criteria for hyperuricemia, ensuring consistency in patient eligibility; (2) Interventions included any of the following antihypertensive agents: allisartan, amlodipine, benazepril, candesartan, captopril, enalapril, felodipine, fosinopril, irbesartan, lisinopril, losartan, nifedipine, perindopril, telmisartan, valsartan, or the combination regimens specified above; (3) Outcomes of interest included changes in serum uric acid (SUA) levels and the “effective rate”, defined as a ≥ 10% reduction in SUA. This threshold was selected because all included trials used it to define treatment effectiveness, reflecting a widely accepted standard in Chinese clinical research. Moreover, a ≥ 10% reduction in SUA is recognized as clinically meaningful in international guidelines and meta-analyses [13]; and (4) Study design was an RCT.

### Data collection and quality assessment

The following items were extracted from each included study: first author’s name, publication year, region, sample size, age, male proportion, SUA level, intervention, control, follow-up duration, and reported outcomes. The methodological quality of the included trials was assessed using the Cochrane Risk of Bias (RoB) 2.0 tool, which evaluates five domains: bias arising from the randomization process, bias due to deviations from intended interventions, bias due to missing outcome data, bias in measurement of the outcome, and bias in selection of the reported result [14]. Data extraction and quality assessments were conducted independently by two authors (HX and DH). Any discrepancies were resolved by a third author (ZY) through referral to the full text of the original articles.

### Statistical analysis

The effective rate (defined as a ≥ 10% reduction in SUA) was assigned as dichotomous data (event: achieved effective rate; non-event: did not achieve effective rate), while changes in SUA levels were analyzed as continuous data. For dichotomous outcomes, we chose the odds ratio (OR) (with 95% confidence intervals [CIs]) as the effect measure, even though only prospective RCTs were included. This choice was based on mathematical stability and consistency in network analysis, approximation to RR in low-event scenarios, and standard practice and software compatibility. A network meta-analysis was conducted to compare and rank the efficacy of different antihypertensive agents in patients with hypertension and hyperuricemia, based on both direct and indirect evidence [15]. Loop-specific analysis (node-splitting analysis) was used to assess the local consistency between direct and indirect estimates for each head-to-head comparison [16]. For node-splitting analysis, we calculated the inconsistency *P*-values for each comparison with both direct and indirect evidence, where a *P*-value >0.05 indicated no significant local inconsistency. The design-by-treatment interaction model was used to assess global inconsistency across the entire network [15]; a *P*-value >0.05 from this model suggested no significant global inconsistency between direct and indirect evidence. The surface under the cumulative ranking curve (SUCRA) was calculated to rank the treatments for each outcome [17]. Pairwise comparison analyses were subsequently performed for all outcomes of interest. Publication bias was evaluated using comparison-adjusted funnel plots, along with Egger’s and Begg tests [18–20]. A two-sided significance level of 0.05 was applied for all statistical tests All analyses were conducted using STATA software (version 12.0; Stata Corporation, College Station, TX, USA).

## Results

### Literature search and study selection

**Fig. 1:**
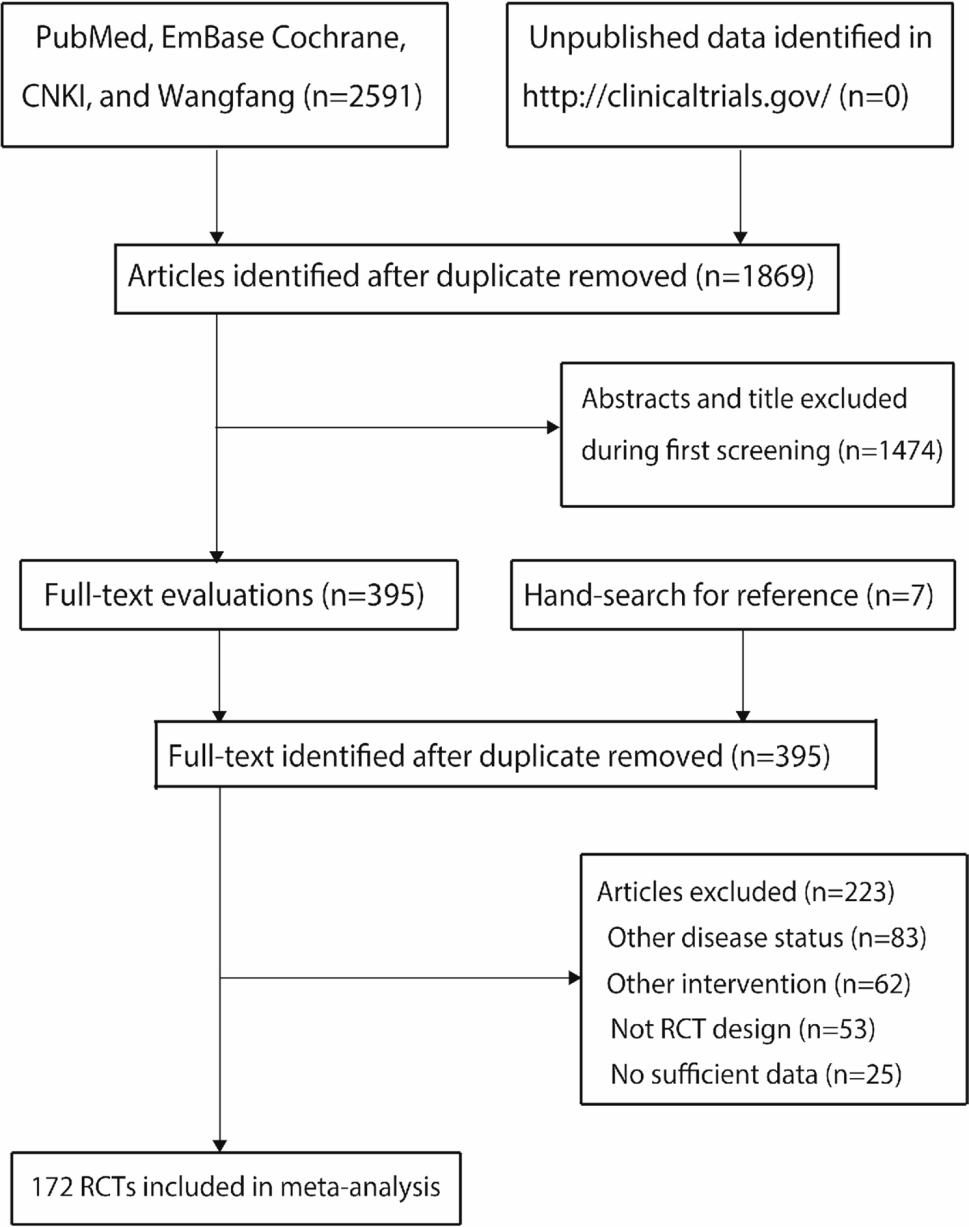
The PRISMA flowchart for literature search and study selection

A total of 2,591 records were identified through the initial electronic search. After removing duplicates, 1,869 studies remained. Among these, 1,474 were excluded due to irrelevance to the topic. The remaining 395 studies underwent full-text assessment. After detailed evaluations, 223 studies were excluded for the following reasons: involving other disease statuses (*n* = 83), testing other interventions (*n* = 62), not being RCTs (*n* = 53), or having insufficient data (*n* = 25). An additional seven potentially eligible trials identified from reference lists of relevant articles were excluded as they had already been captured in the electronic search. Ultimately, 172 RCTs were included in the final analysis. The study selection process is illustrated in Fig. 1.

### Study characteristics

The baseline characteristics of the included trials and participants are summarized in Supplemental File 2. A total of 16,226 hypertensive patients with hyperuricemia were included, with sample sizes ranging from to 31–325. The mean age of participants ranged from 44.0 to 85.9 years, and the follow-up duration varied from 2 to 48 weeks. The methodological quality of the included trials, assessed as described in Supplemental File 3, was generally low to moderate quality, with most studies exhibiting a moderate to high risk of bias.

### Changes in SUA

**Fig. 2:**
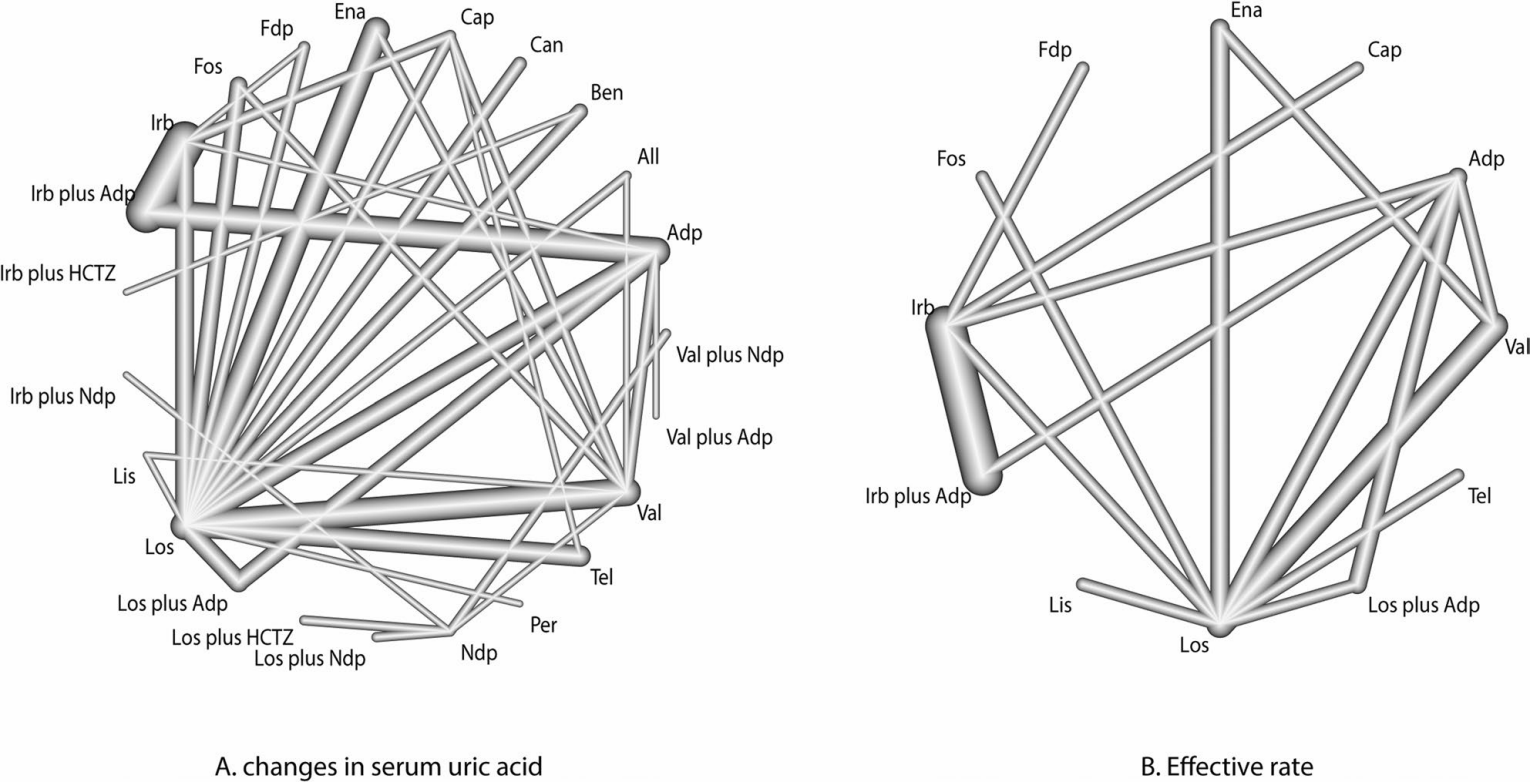
Network of comparisons for the reduction in serum uric acid (**A**) and effective rate (**B**) included in the analysis. Nodes represent treatment methods: Each node represents a specific treatment method or intervention. The size of the node typically reflects the sample size or weight of that treatment method in the analysis. Edges represent comparisons: Edges represent comparisons between two treatment methods. The width or style of the edge can indicate the number or weight of comparisons. Connections between nodes: Connections between edges represent the feasibility of direct comparisons. For example, if a study compares A with B, and B with C, there will be a connection from A to C, indicating the existence of an indirect comparison between A and C. Node placement: Node placement may be arranged based on certain attributes, such as the similarity of treatment methods or according to their effect sizes. Effect size and uncertainty: The width or style of the edges between nodes is typically adjusted based on the effect size and uncertainty of the comparison. Wider or more prominent lines may indicate larger effects or greater certainty. All: allisartan; Adp: amlodipine; Ben: benazepril; Can: candesartan; Cap: captopril; Ena: enalapril; Fdp: felodipine; Fos: fosinopril; HCTZ: hydrochlorothiazide; Irb: irbesartan; Lis: lisinopril; Los: losartan; Ndp: nifedipine; Per: perindopril; Tel: telmisartan; Val: valsartan

**Fig. 3:**
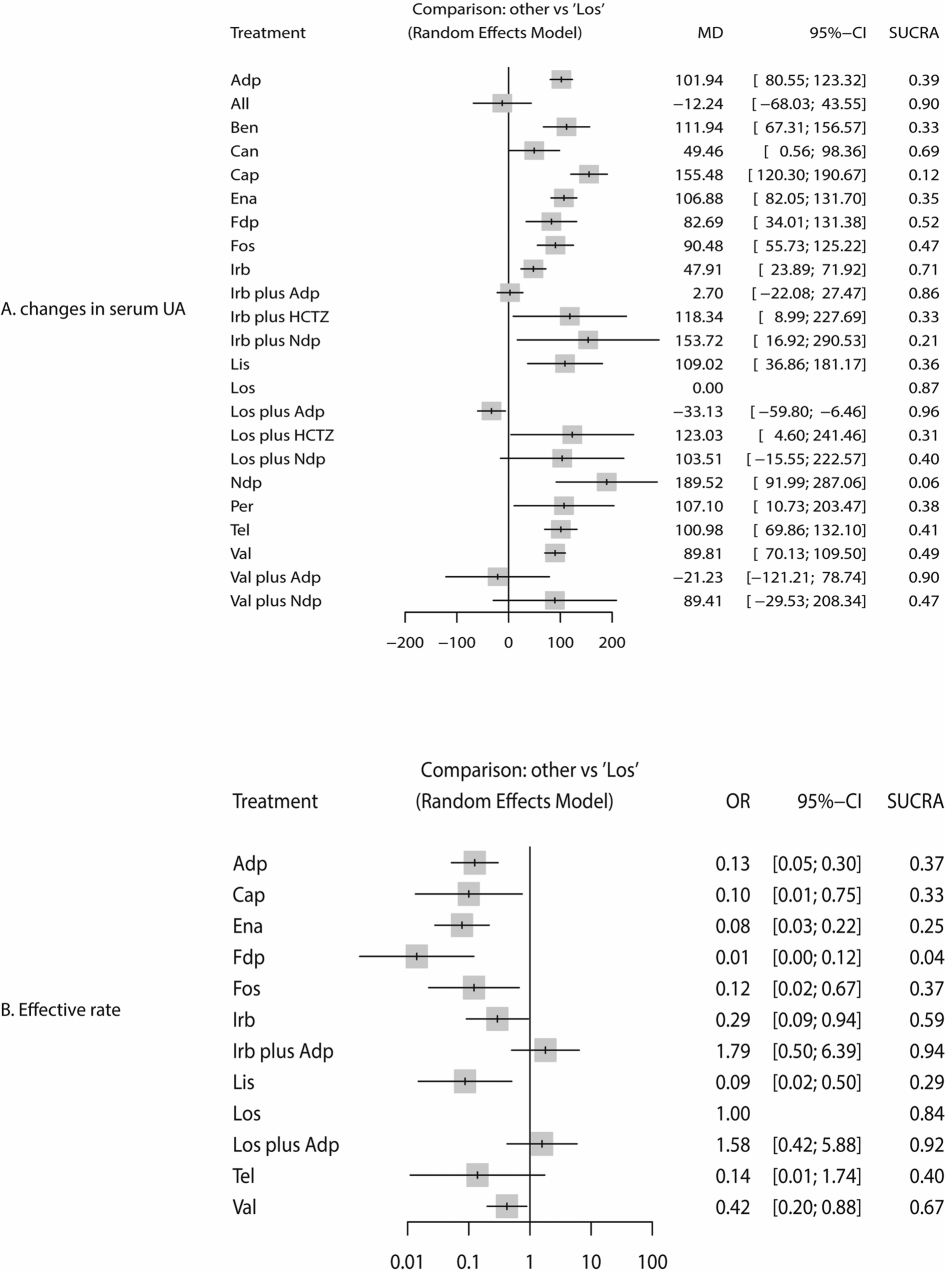
The Forest plot for the reduction in serum uric acid (**A**) and effective rate (**B**). All: allisartan; Adp: amlodipine; Ben: benazepril; Can: candesartan; Cap: captopril; CI: confidence interval; Ena: enalapril; Fdp: felodipine; Fos: fosinopril; HCTZ: hydrochlorothiazide; Irb: irbesartan; Lis: lisinopril; Los: losartan; MD: mean difference; Ndp: nifedipine; OR: odds ratio; Per: perindopril; SUCRA: surfaces under the cumulative ranking; Tel: telmisartan; Val: valsartan

**Fig. 4:**
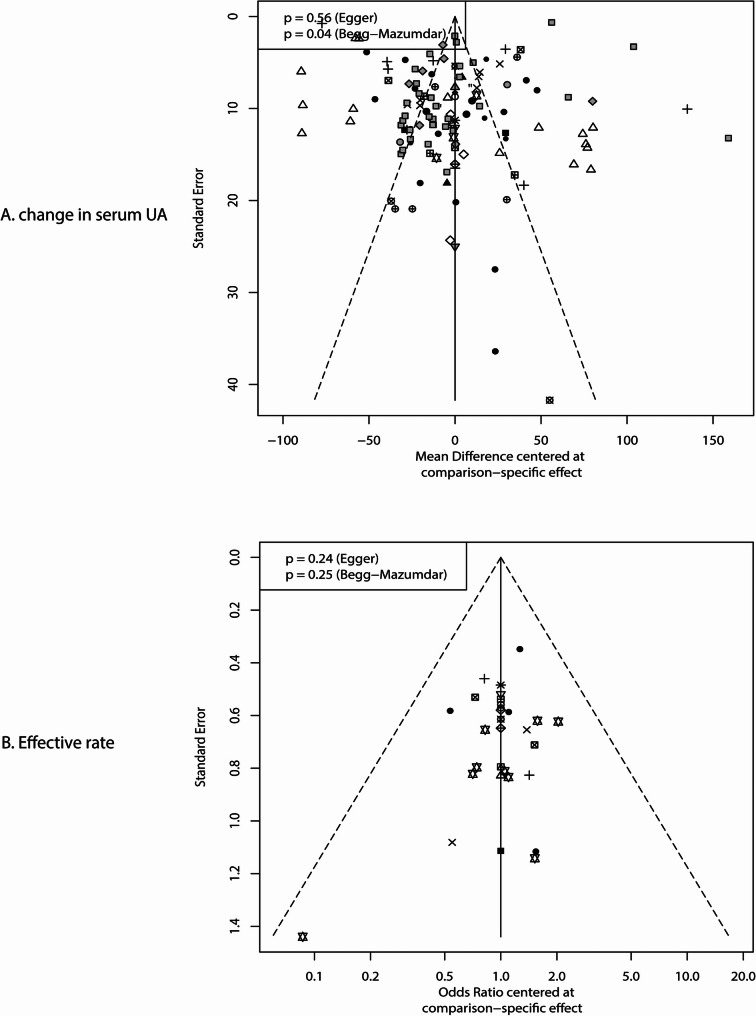
Funnel plot for the reduction in serum uric acid (**A**) and effective rate (**B**)

The network of comparisons for changes in SUA is shown in Fig. 2A. The size of nodes corresponds to the number of trials for each treatment, and the thickness of edges reflects the precision of direct estimates. Using SUCRA probabilities to rank the treatments, losartan plus amlodipine (SUCRA: 96%), valsartan plus amlodipine (SUCRA: 90%), and allisartan (SUCRA: 90%) demonstrated superior efficacy in reducing SUA levels (Fig. 3A). When losartan was used as the reference, amlodipine, benazepril, candesartan, captopril, enalapril, felodipine, fosinopril, irbesartan, irbesartan plus HCTZ, irbesartan plus nifedipine, lisinopril, losartan plus HCTZ, nifedipine, perindopril, telmisartan, and valsartan were associated with higher SUA levels, while losartan plus amlodipine was more effective than losartan alone (Fig. 3A). Detailed results of pairwise comparisons are provided in Supplemental File 4. Significant differences in SUA levels were observed for the following comparisons: benazepril vs. losartan (WMD: 111.94 µmol/L; 95% CI: 67.31 to 156.57), candesartan vs. losartan (WMD: 49.46 µmol/L; 95% CI: 0.56 to 98.36), captopril vs. irbesartan (WMD: 107.58 µmol/L; 95% CI: 70.31 to 144.85), captopril vs. losartan (WMD: 155.48 µmol/L; 95% CI: 120.30 to 190.67), captopril vs. telmisartan (WMD: 54.50 µmol/L; 95% CI: 10.19 to 98.82), captopril vs. valsartan (WMD: 65.67 µmol/L; 95% CI: 28.11 to 103.22), enalapril vs. losartan (WMD: 106.88 µmol/L; 95% CI: 82.05 to 131.70), felodipine vs. losartan (WMD: 82.69 µmol/L; 95% CI: 34.01 to 131.38), fosinopril vs. losartan (WMD: 90.48 µmol/L; 95% CI: 55.73 to 125.22), irbesartan vs. irbesartan plus amlodipine (WMD: 45.21 µmol/L; 95% CI: 29.47 to 60.95), irbesartan vs. losartan (WMD: 47.91 µmol/L; 95% CI: 23.89 to 71.92), and lisinopril vs. losartan (WMD: 109.02 µmol/L; 95% CI: 36.86 to 181.17). Begg’s test yielded a significant result (*P* = 0.04), suggesting potential publication bias, while Egger’s test was non-significant (*P* = 0.56), providing no strong evidence of bias. This discrepancy is common in meta-analyses and likely reflects the tests’ differing sensitivities: Begg’s test is more sensitive to small-study effects, while Egger’s test is more robust to outliers but may have lower power in networks with heterogeneous study sizes. Critically, the significant Begg’s test cannot be ignored: it constitutes statistical evidence suggestive of potential publication bias for the mean SUA change outcome, even if Egger’s test did not confirm this. The comparison-adjusted funnel plot visually supports this ambiguity, with mild asymmetry that aligns with the conflicting statistical test results (Fig. 4A).

### Effective rate

The network of comparisons for the effective rate is presented in Fig. 2B. Based on SUCRA values, irbesartan plus amlodipine (SUCRA: 94%), losartan plus amlodipine (SUCRA: 92%), and losartan monotherapy (SUCRA: 84%) were associated with higher effective rates (Fig. 3B). When losartan was used as the reference, amlodipine, captopril, enalapril, felodipine, fosinopril, irbesartan, lisinopril, and valsartan were associated with lower effective rates (Fig. 3B). Key results from pairwise comparisons (Supplemental File 4) include: amlodipine vs. irbesartan plus amlodipine (OR: 0.07; 95% CI: 0.02–0.23), amlodipine vs. losartan (OR: 0.13; 95% CI: 0.05–0.30), amlodipine vs. losartan plus amlodipine (OR: 0.08; 95% CI: 0.02–0.27), amlodipine vs. valsartan (OR: 0.30; 95% CI: 0.11–0.83), enalapril vs. losartan (OR: 0.08; 95% CI: 0.03–0.22), enalapril vs. valsartan (OR: 0.18; 95% CI: 0.06–0.55), felodipine vs. irbesartan (OR: 0.05; 95% CI: 0.01–0.29), fosinopril vs. losartan (OR: 0.12; 95% CI: 0.02–0.67), irbesartan vs. irbesartan plus amlodipine (OR: 0.16; 95% CI: 0.09–0.32), irbesartan vs. losartan (OR: 0.29; 95% CI: 0.09–0.94), lisinopril vs. losartan (OR: 0.09; 95% CI: 0.02–0.50), and valsartan vs. losartan (OR: 0.42; 95% CI: 0.20–0.88). No significant publication bias was detected for the effective rate outcomes (Egger’s test *P* = 0.24; Begg’s test *P* = 0.25; Fig. 4B).

### Consistency assessment

To verify the reliability of the network meta-analysis results, we conducted both global and local inconsistency assessments. For global inconsistency, the design-by-treatment interaction model yielded a *P*-value of 0.32 (for changes in SUA levels) and 0.41 (for effective rate), respectively. Since both *P*-values were greater than 0.05, there was no significant global inconsistency across the entire network, indicating that the overall consistency between direct and indirect evidence was acceptable. For local inconsistency, node-splitting analysis was performed for all comparisons that had both direct and indirect evidence. The results showed that the inconsistency *P*-values for all these comparisons ranged from 0.15 to 0.78 (for changes in SUA levels) and 0.12 to 0.83 (for effective rate), with none of the *P*-values < 0.05. This indicated that there was no significant local inconsistency between direct and indirect evidence for individual comparisons.

## Discussion

This systematic review and network meta-analysis is more comprehensive, having included 22 different treatment regimens and evaluated changes in SUA levels and the effective rate. The present comprehensive quantitative analysis identified 172 RCTs involving 16,226 hypertensive patients with hyperuricemia who were randomly assigned to receive 22 different treatment regimens. The study found that greater reductions in SUA levels were achieved with losartan plus amlodipine, valsartan plus amlodipine, and allisartan. Moreover, the use of irbesartan plus amlodipine, losartan plus amlodipine, and losartan was associated with a higher effective rate. This study is the first to compare various antihypertensive agents in terms of uric acid-lowering effects in hypertensive patients with hyperuricemia, and the results should be considered in clinical practice for this patient population.

Direct international evidence regarding antihypertensive agents for hypertension with hyperuricemia remains limited, as most high-quality non-Chinese studies focus solely on hypertensive patients. Furthermore, due to the lack of international data on ARB (angiotensin II receptor blocker) and amlodipine combinations, the external validity of these findings to non-Chinese populations must be interpreted with caution. In addition, all included RCTs were consistently of low quality, with a high risk of bias concentrated in three domains: bias due to deviations from intended interventions, bias in measurement of the outcome, and bias in selection of the reported result. Finally, unlike traditional meta-analysis, network meta-analysis evaluates consistency between direct and indirect evidence—the primary measure of heterogeneity in such analyses, including loop-specific inconsistency tests and the design-by-treatment interaction model. In our study, the results of these consistency assessments showed no significant global or local inconsistency, confirming that the direct and indirect evidence were mutually consistent and thus enhancing the reliability of our efficacy ranking conclusions.

The superior efficacy of ARB + amlodipine combinations (e.g., losartan + amlodipine, valsartan + amlodipine) over ARB monotherapies in reducing SUA stems from synergistic biological mechanisms that targete both uric acid excretion and production, as well as improve renal function [11]. ARBs (e.g., losartan, valsartan) exert uricosuric effects primarily by inhibiting URAT1-mediated urate reabsorption in the proximal tubule, thereby enhancing uric acid excretion [21, 22]. Moreover, the beneficial effects of allisartan can be attributed to its complete hydrolysis to EX P3174 by esterases during oral absorption in vivo, which selectively binds to the angiotensin II type 1 (AT1) receptor without potentiating bradykinin-mediated effects. Alisartan is a prodrug of EXP3174, the active metabolite of losartan. The amount of EXP3174 produced by alisartan is 41 times greater than that produced by losartan potassium, and its antihypertensive effect is significantly stronger than that of losartan [23]. Additionally, ARBs suppress the renin-angiotensin system (RAS), which reduces renal vasoconstriction and improves glomerular filtration rate, thereby indirectly facilitating uric acid clearance [24]. The central role of the RAS in hypertension is further underscored by research showing that genetic variations in the ACE gene can interact to influence susceptibility to essential hypertension [25]. Moreover, the key enzyme ACE, which is targeted by many antihypertensive drugs, not only generates the potent vasoconstrictor Angiotensin II but also inactivates vasodilators, making it a critical focus for therapeutic intervention [26]. The pathological effects of excessive Ang II, such as increased blood pressure variability and vascular remodeling, have been directly demonstrated in animal models and can be ameliorated by calcium channel blockers like azelnidipine [27]. However, ARB monotherapy may be limited in some patients due to residual urate reabsorption or insufficient modulation of renal hemodynamics.

Amlodipine, a dihydropyridine calcium channel blocker, complements ARBs through distinct yet synergistic pathways. It inhibits purine nucleotide synthesis by downregulating key enzymes in the purine metabolic pathway, thereby reducing de novo uric acid production [28, 29]. Furthermore, amlodipine improves renal tubular function by attenuating tubular injury and oxidative stress, which are common in hypertensive patients with hyperuricemia [30]. This tubular protection enhances the uricosuric effect of ARBs by preserving the integrity of the proximal tubule, where URAT1-mediated reabsorption occurs.

Critically, the combination acts synergistically at the renal level: the vasodilatory effect of amlodipine on afferent arterioles increases renal blood flow, which potentiates ARB-mediated RAS inhibition. This dual action further reduces renal vasoconstriction, enhances GFR, and creates a more favorable tubular environment for uric acid excretion. Moreover, amlodipine could upregulate the expression of ABCG2 (a urate efflux transporter) in renal tubular cells, working in concert with ARB-mediated URAT1 inhibition to reduce net urate reabsorption. Collectively, ARBs primarily reduce uric acid reabsorption, while amlodipine reduces uric acid production and improves renal function. Their combined effects create a multi-targeted approach that exceeds the efficacy of ARB monotherapy in lowering SUA.

The effective rate was defined as a reduction in SUA of at least 10%. A number of the included studies reported the uric acid-lowering effect of various antihypertensive agents in hypertensive patients with hyperuricemia. The results of this study revealed that irbesartan plus amlodipine, losartan plus amlodipine, and losartan monotherapy had relatively better effects on the effective rate. The uricosuric action of losartan occurs through inhibition of URAT1. Therefore, patients with hypouricemia harboring URAT1 mutations did not exhibit an SUA-lowering effect when treated with losartan [21]. A previous study demonstrated that the URAT1 inhibitory effects of irbesartan were superior to those of losartan in vitro and that the SUA-lowering effect of irbesartan was mediated by increased urinary uric acid excretion [31].

Several limitations of this study should be mentioned. (1) A key limitation is that all included RCTs were conducted in China, with no eligible trials identified from non-Chinese populations. This geographic clustering directly limits the generalizability of our findings to non-Chinese individuals, as SUA metabolism and responses to antihypertensive drugs vary substantially across regions. Despite repeated searches, no additional non-Chinese RCTs could be identified, indicating that this is a practical limitation of the current evidence base rather than a methodological oversight. (2) Another practical limitation is the limited accessibility of the included studies. Many RCTs were published in Chinese-language journals that are not indexed in international databases or are only available on Chinese national platforms. As a result, some studies cannot be retrieved via Google Scholar or international library portals, making independent verification of the reported data challenging for readers outside China. (3) Most of the included trials were of low quality and reported a high risk bias for bias due to deviations from intended interventions, bias in measurement of the outcome, and bias in selection of the reported result. (4) The severity of hypertension and hyperuricemia varied across the included trials, and it was not possible to conduct subgroup or sensitivity analyses based on baseline severity due to systematically missing or inconsistently reported data. (5) Background therapies (including dietary and drug interventions) differed among patients, which could affect the prognosis of hypertensive patients with hyperuricemia. (6) The doses of antihypertensive agents differed among the included trials, which may have influenced the changes in SUA. (7) We did not conduct a traditional subgroup analysis stratified by follow-up duration (2–48 weeks). This decision was based on the unique constraints of the network meta-analysis design, which differs from standard pairwise meta-analysis. (8) The inability to fully assess the transitivity assumption—critical for network meta-analysis validity—due to inconsistent reporting of baseline and study design characteristics across included trials. (9) There are inherent limitations to meta-analysis based on published articles, including inevitable publication bias and restricted detailed analysis.

## Conclusion

This systematic review and network meta-analysis provides moderate-certainty evidence regarding the relative efficacy of antihypertensive regimens in Chinese patients with hypertension and hyperuricemia. For SUA reduction, losartan plus amlodipine, valsartan plus amlodipine, and allisartan monotherapy emerged as some of the most effective regimens, based on SUCRA rankings and pairwise effect sizes. For achieving a clinically meaningful reduction in SUA (≥ 10%, defined as the effective rate), irbesartan plus amlodipine, losartan plus amlodipine, and losartan monotherapy showed the highest relative efficacy. However, these findings must be interpreted with caution due to important limitations. Future large-scale, high-quality RCTs—ideally multicenter, international, and with long-term follow-up—are needed to validate these findings and assess the clinical relevance of SUA reduction for cardiovascular and renal outcomes.

## Supplementary Information

Supplementary Material 1.

Supplementary Material 2.

Supplementary Material 3.

Supplementary Material 4.

## Data Availability

The datasets used and/or analysed during the current study are available from the corresponding author on reasonable request.
