# Supplementary material for: Antihypertensive drugs for hyperuricemia in patients with hypertension: a systematic review and network meta-analysis of Chinese trials
Source: BMC Cardiovasc Disord. 2025 Dec 2;25:856. doi: 10.1186/s12872-025-05339-7 (PMC12673777; doi:10.1186/s12872-025-05339-7)
Supplement: Supplementary file 1 — Supplementary Material 1. [file 12872_2025_5339_MOESM1_ESM.docx]

**PubMed:** (Hypertension [MeSH] OR "blood pressure lowering drugs" OR allisartan OR amlodipine OR benazepril OR candesartan OR captopril OR enalapril OR felodipine OR fosinopril OR irbesartan OR lisinopril OR losartan OR nifedipine OR perindopril OR telmisartan OR valsartan OR "irbesartan plus amlodipine" OR "irbesartan plus hydrochlorothiazide" OR "irbesartan plus nifedipine" OR "losartan plus amlodipine" OR "losartan plus nifedipine" OR "valsartan plus amlodipine" OR "valsartan plus nifedipine") AND (Hyperuricemia [MeSH] OR "uric acid" OR "urate") AND ("Randomized Controlled Trials as Topic" [MeSH] OR "RCT") AND ("humans" [MeSH]).

**EmBase:** (("Hypertension"[Emtree] OR "Antihypertensive Agents"[Emtree] OR "blood pressure lowering drugs" OR "antihypertensive drugs" OR "hypertensive" OR allisartan OR amlodipine OR benazepril OR candesartan OR captopril OR enalapril OR felodipine OR fosinopril OR irbesartan OR lisinopril OR losartan OR nifedipine OR perindopril OR telmisartan OR valsartan OR "irbesartan plus amlodipine" OR "irbesartan plus hydrochlorothiazide" OR "irbesartan plus nifedipine" OR "losartan plus amlodipine" OR "losartan plus nifedipine" OR "valsartan plus amlodipine" OR "valsartan plus nifedipine") ) AND (("Hyperuricaemia"[Emtree] OR "Uric Acid"[Emtree] OR "urate" OR "hyperuricemia" OR "serum uric acid") ) AND (("Randomized Controlled Trial"[Emtree] OR "RCT" OR "randomised controlled trial" OR "randomized trial") ) AND ("Humans"[Emtree]).

**Cochrane library:** (("Hypertension"[MeSH Terms] OR "hypertension":ti,ab,kw OR "Antihypertensive Agents"[MeSH Terms] OR "antihypertensive agents":ti,ab,kw OR "blood pressure lowering drugs":ti,ab,kw) AND ("Hyperuricemia"[MeSH Terms] OR "hyperuricemia":ti,ab,kw OR "Hyperuricaemia"[MeSH Terms] OR "hyperuricaemia":ti,ab,kw OR "Uric Acid"[MeSH Terms] OR "uric acid":ti,ab,kw OR "urate":ti,ab,kw) AND ("Randomized Controlled Trials"[MeSH Terms] OR "randomized controlled trial":ti,ab,kw OR "RCT":ti,ab,kw OR "randomised controlled trial":ti,ab,kw) AND ("Humans"[MeSH Terms] OR "humans":ti,ab,kw))

**Chinese National Knowledge Infrastructure/Wanfang:** （主题：高血压 OR 抗高血压药 OR 阿利沙坦 OR 氨氯地平 OR 贝那普利 OR 坎地沙坦 OR 卡托普利 OR 依那普利 OR 非洛地平 OR 福辛普利 OR 厄贝沙坦 OR 赖诺普利 OR 氯沙坦 OR 硝苯地平 OR 培哚普利 OR 替米沙坦 OR 缬沙坦 OR 厄贝沙坦+氨氯地平 OR 厄贝沙坦+氢氯噻嗪 OR 厄贝沙坦+硝苯地平 OR 氯沙坦+氨氯地平 OR 氯沙坦+硝苯地平 OR 缬沙坦+氨氯地平 OR 缬沙坦+硝苯地平）AND（主题：高尿酸血症 OR 尿酸）AND（文献类型：随机对照试验）
