# Supplementary material for: Antihypertensive drugs for hyperuricemia in patients with hypertension: a systematic review and network meta-analysis of Chinese trials
Source: BMC Cardiovasc Disord. 2025 Dec 2;25:856. doi: 10.1186/s12872-025-05339-7 (PMC12673777; doi:10.1186/s12872-025-05339-7)
Supplement: Supplementary file 2 — Supplementary Material 2. [file 12872_2025_5339_MOESM2_ESM.docx]

Table S1. The baseline characteristics of included trials and involved patients

| Study | Region | Sample size | Age (years) | Male (%) | Serum UA (μmol/L) | Definition of hyperuricemia | Intervention | Control | Follow-up |
| --- | --- | --- | --- | --- | --- | --- | --- | --- | --- |
| Wang 2000 [1] | China | 65 | 72.4 | 69.2 | 560 | 500-630 μmol/L | Los | Fos | 8 weeks |
| Zhang 2001 [2] | China | 100 | 65.0 | 62.0 | 540 | > 420 μmol/L | Los | Cap | 4 weeks |
| Yang 2001 [3] | China | 80 | 67.9 | 78.8 | 410 | > 420 μmol/L | Val | Ena | 2 weeks |
| Xu 2001 [4] | China | 80 | 57.7 | 67.5 | 430 | > 420 μmol/L | Los | Ena | 2 weeks |
| Ma 2002 [5] | China | 60 | 58.0 | 73.3 | 430 | > 420 μmol/L | Los | Ben | 3 weeks |
| Chen 2003 [6] | China | 65 | 44.0 | 58.5 | 420 | NA | Irb | Cap | 4 weeks |
| Nie 2003 [7] | China | 58 | 56.2 | 65.5 | 570 | Male: > 420 μmol/L; female: > 357 μmol/L | Los | Ben | 4 weeks |
| Xu 2004 [8] | China | 60 | 66.2 | 36.7 | 370 | Male: > 416 μmol/L; female: > 339 μmol/L | Los | Fdp | 8 weeks |
| Zhang 2005 [9] | China | 60 | 69.5 | 55.0 | 510 | 416-720 μmol/L | Los | Cap | 4 weeks |
| Zhang 2005 [10] | China | 61 | 71.5 | 54.1 | 450 | 330-575 μmol/L | Los | Lis | 8 weeks |
| Dang 2006 [11] | China | 325 | 57.1 | 57.8 | 430 | Male: > 406 μmol/L; female: > 310 μmol/L | Los | Irb | 4 weeks |
| Zhang 2006 [12] | China | 68 | 60.0 | 75.0 | 490 | ≥ 417 μmol/L | Los | Adp | 16 weeks |
| Zhu 2006 [13] | China | 86 | 56.9 | 72.1 | 440 | 410-530 μmol/L | Los | Fos | 8 weeks |
| Du 2006 [14] | China | 71 | 59.0 | 69.0 | 430 | Male: > 390 μmol/L; female: > 350 μmol/L | Los | Ena | 4 weeks |
| Zheng 2006 [15] | China | 68 | 46.0 | 58.8 | 720 | Male: > 420 μmol/L; female: > 340 μmol/L | Los | Cap | 4 weeks |
| Liu 2006 [16] | China | 98 | 66.2 | 49.0 | 460 | NA | Los | Adp | 8 weeks |
| Li 2006 [17] | China | 44 | 62.8 | 56.8 | 530 | > 420 μmol/L | Los | Ena | 4 weeks |
| Fan 2006 [18] | China | 60 | 61.4 | 73.3 | 430 | NA | Los | Ben | 4 weeks |
| Chen 2007 [19] | China | 160 | 64.6 | 80.0 | 410 | > 420 μmol/L | Val | Cap | 2 weeks |
| Zhang 2008 [20] | China | 82 | 50.3 | 58.5 | 510 | Male: > 390 μmol/L; female: > 310 μmol/L | Los | Adp | 12 weeks |
| Gu 2008 [21] | China | 100 | 33-70 | NA | 440 | 410-530 μmol/L | Los | Adp | 8 weeks |
| Zhao 2008 [22] | China | 86 | 58.2 | 83.7 | 480 | Male: > 420 μmol/L; female: > 360 μmol/L | Irb | Cap | 8 weeks |
| Yuan 2008 [23] | China | 86 | 42-76 | 53.5 | 500 | Male: > 420 μmol/L; female: > 360 μmol/L | Val | Adp | 8 weeks |
| Qi 2008 [24] | China | 66 | NA | 68.2 | 460 | NA | Los | Ena | 6 weeks |
| Chen 2008 [25] | China | 68 | 66.9 | 69.1 | 520 | NA | Val | Cap | 8 weeks |
| Zeng 2009 [26] | China | 104 | 34-84 | 53.8 | 490 | 410-556 μmol/L | Los | Adp | 8 weeks |
| Yuan 2009 [27] | China | 110 | 63.2 | 55.5 | 520 | Male: > 420 μmol/L; female: > 350 μmol/L | Los | Ena | 4 weeks |
| Zhang 2009 [28] | China | 31 | 66.7 | 71.0 | 520 | Male: > 420 μmol/L; female: > 360 μmol/L | Los + Adp | Los | 4 weeks |
| Jiang 2009 [29] | China | 85 | 65.0 | 70.6 | 430 | Male: > 420 μmol/L; female: > 350 μmol/L | Los | Val | 8 weeks |
| Li 2009 [30] | China | 80 | 58.2 | 62.5 | 490 | Male: > 416 μmol/L; female: > 386 μmol/L | Los | Ena | 8 weeks |
| Cai 2009 [31] | China | 72 | 64.8 | 61.1 | 470 | Male: > 390 μmol/L; female: > 310 μmol/L | Los | Tel | 8 weeks |
| Liao 2009 [32] | China | 88 | NA | 73.9 | 460 | NA | Los | Fos | 6 weeks |
| Cheng 2010 [33] | China | 135 | 64.6 | 78.5 | 420 | NA | Tel | Cap | 2 weeks |
| Tian 2010 [34] | China | 90 | 54.6 | 60.0 | 450 | Male: > 416 μmol/L; female: > 386 μmol/L | Los | Irb | 12 weeks |
| Wang 2010 [35] | China | 76 | 55.8 | 60.5 | 460 | NA | Val | Adp | 8 weeks |
| Gong 2010 [36] | China | 110 | NA | 60.9 | 480 | Male: > 420 μmol/L; female: > 360 μmol/L | Val | Lis | 6 weeks |
| Wang 2010 [37] | China | 72 | 45-78 | 45.8 | NA | Male: > 420 μmol/L; female: > 360 μmol/L | Irb | Fdp | 8 weeks |
| Zhang 2010 [38] | China | 186 | NA | 56.5 | 470 | 407-0507 μmol/L | Val | Fos | 8 weeks |
| Xie 2010 [39] | China | 80 | 51.6 | 65.0 | 460 | 410-520 μmol/L | Los | Fos | 8 weeks |
| Zhang 2011 [40] | China | 112 | 85.9 | 59.8 | 550 | Male: > 416 μmol/L; female: > 357 μmol/L | Irb + Adp | Irb, Adp | 24 weeks |
| Li 2011 [41] | China | 77 | 66.1 | 53.2 | 430 | Male: > 390 μmol/L; female: > 310 μmol/L | Los | Tel | 8 weeks |
| Wu 2011 [42] | China | 120 | 69.5 | 59.2 | 480 | Male: > 420 μmol/L; female: > 350 μmol/L | Los | Ena | 8 weeks |
| He 2011 [43] | China | 100 | 50.1 | 58.0 | 490 | Male: > 420 μmol/L; female: > 360 μmol/L | Val | Ena | 4 weeks |
| Zeng 2011 [44] | China | 70 | 58.2 | 50.0 | 490 | NA | Los | Irb | 8 weeks |
| Xia 2011 [45] | China | 110 | 52.1 | 62.7 | 440 | 420-520 μmol/L | Los | Fos | 8 weeks |
| Wu 2012 [46] | China | 60 | 66.1 | 73.3 | 480 | Male: > 420 μmol/L; female: > 357 μmol/L | Los | Val | 8 weeks |
| Tian 2012 [47] | China | 86 | 50.4 | 55.8 | 450 | Male: > 416 μmol/L; female: > 386 μmol/L | Los | Fos | 24 weeks |
| Lu 2012 [48] | China | 190 | 64.3 | 67.9 | 530 | NA | Los + Adp | Los | 4 weeks |
| Li 2012 [49] | China | 82 | 53.4 | 72.0 | 470 | NA | Los | Per | 12 weeks |
| Shao 2012 [50] | China | 134 | 56.6 | 53.7 | 440 | Male: > 420 μmol/L; female: > 360 μmol/L | Irb | Cap | 8 weeks |
| Zhao 2013 [51] | China | 114 | 69.0 | 54.4 | 490 | NA | Los | Tel | 8 weeks |
| Wang 2013 [52] | China | 60 | 56.2 | 60.0 | 430 | NA | Los + Adp | Los | 4 weeks |
| Tang 2013 [53] | China | 40 | 67.0 | 71.5 | 550 | > 420 μmol/L | Los | Adp | 4 weeks |
| Zhang 2013 [54] | China | 173 | 71.0 | 37.6 | NA | Male: > 420 μmol/L; female: > 380 μmol/L | Los | Val | 12 weeks |
| Xu 2013 [55] | China | 60 | 30-70 | NA | 460 | > 430 μmol/L | Irb + HCTZ | Ben | 12 weeks |
| Song 2014 [56] | China | 68 | 66.3 | 67.6 | 440 | 420-520 μmol/L | Los | Fdp | 6 weeks |
| Zhang 2014 [57] | China | 140 | 74.9 | 55.7 | 560 | NA | Irb + Adp | Irb | 24 weeks |
| Wang 2014 [58] | China | 71 | 69.0 | 60.6 | 560 | Male: > 416 μmol/L; female: > 357 μmol/L | Irb + Adp | Adp | 12 weeks |
| Fang 2014 [59] | China | 120 | 58.4 | 61.7 | 450 | Male: > 420 μmol/L; female: > 360 μmol/L | Irb | Adp | 12 weeks |
| Zhong 2014 [60] | China | 70 | 62.2 | 57.1 | 430 | NA | Los + Adp | Adp | 4 weeks |
| Wang 2014 [61] | China | 60 | 66.0 | 58.3 | 430 | NA | Val | Adp | 8 weeks |
| Yao 2014 [62] | China | 66 | 64.5 | 69.7 | 430 | Male: > 420 μmol/L; female: > 350 μmol/L | Los | Ben | 8 weeks |
| Yang 2014 [63] | China | 100 | 66.4 | 66.0 | NA | Male: > 420 μmol/L; female: > 380 μmol/L | Los | Val | 3 weeks |
| Chen 2014 [64] | China | 123 | 74.4 | 58.5 | 550 | NA | Irb + Adp | Irb, Adp | 24 weeks |
| Meng 2014 [65] | China | 80 | 60.2 | 53.8 | 520 | NA | Los | Ena | 4 weeks |
| Tang 2014 [66] | China | 110 | 62.3 | 55.5 | 520 | Male: > 420 μmol/L; female: > 350 μmol/L | Los | Ena | 4 weeks |
| Fang 2014 [67] | China | 82 | 65.9 | 52.4 | 490 | Male: > 420 μmol/L; female: > 265 μmol/L | Los | Ena | 4 weeks |
| Yan 2015 [68] | China | 76 | 74.5 | 55.3 | 550 | Male: > 416 μmol/L; female: > 357 μmol/L | Irb + Adp | Irb | 24 weeks |
| Wei 2015 [69] | China | 92 | 68.5 | 62.0 | 560 | NA | Irb + Adp | Adp | 24 weeks |
| Cui 2015 [70] | China | 87 | 71.6 | 77.0 | 550 | NA | Irb + Adp | Irb, Adp | 40 weeks |
| Zhang 2015 [71] | China | 88 | 60.6 | 63.6 | 450 | Male: > 420 μmol/L; female: > 360 μmol/L | Los | Ena | 8 weeks |
| Sun 2015 [72] | China | 92 | 74.3 | 62.0 | 550 | NA | Irb + Adp | Irb | 24 weeks |
| Lu 2015 [73] | China | 78 | 72.9 | 57.7 | NA | NA | Irb + Adp | Irb | 24 weeks |
| Zhang 2015 [74] | China | 108 | 69.6 | 54.6 | 570 | NA | Irb + Adp | Irb | 24 weeks |
| Wang 2015 [75] | China | 278 | 68.4 | 52.5 | 430 | NA | Los | Adp | 8 weeks |
| Yuan 2015 [76] | China | 80 | 52.8 | 76.3 | 490 | > 416 μmol/L | Los | Can | 8 weeks |
| Liao 2015 [77] | China | 86 | 70.4 | 57.0 | 550 | Male: > 416 μmol/L; female: > 357 μmol/L | Irb + Adp | Irb | 24 weeks |
| Shi 2015 [78] | China | 100 | 66.4 | 53.0 | 550 | NA | Irb + Adp | Adp | 24 weeks |
| Li 2015 [79] | China | 120 | 65.0 | 54.2 | 430 | NA | Los + Adp | Adp | 4 weeks |
| Ji 2015 [80] | China | 88 | 69.6 | 58.0 | NA | NA | Irb + Adp | Irb | 4 weeks |
| Zuo 2015 [81] | China | 70 | 77.0 | 52.9 | 470 | NA | Los | Ena | 4 weeks |
| Zhong 2015 [82] | China | 80 | 73.6 | 58.8 | 560 | Male: > 415 μmol/L; female: > 356 μmol/L | Irb + Adp | Irb | 24 weeks |
| Dong 2015 [83] | China | 87 | 85.7 | 65.5 | 550 | NA | Irb + Adp | Irb | 24 weeks |
| Wu 2015 [84] | China | 120 | 85.9 | 65.0 | 550 | > 416 μmol/L | Irb + Adp | Irb | 24 weeks |
| Jin 2015 [85] | China | 88 | 62.3 | 56.8 | 490 | NA | Los | Tel | 8 weeks |
| Li 2015 [86] | China | 96 | 64.0 | 59.4 | 550 | Male: > 416 μmol/L; female: > 357 μmol/L | Irb + Adp | Adp | 24 weeks |
| Li 2015 [87] | China | 76 | 54.2 | 53.9 | 430 | > 420 μmol/L | Val + Ndp | Ndp | 24 weeks |
| Zhong 2015 [88] | China | 90 | 78.8 | 57.8 | NA | NA | Irb + Adp | Irb, Adp | 24 weeks |
| Zhang 2015 [89] | China | 120 | 61.8 | 76.7 | 500 | > 416 μmol/L | Val | Ndp | 12 weeks |
| Han 2015 [90] | China | 32 | 22-65 | 43.8 | 430 | Male: > 420 μmol/L; female: > 360 μmol/L | Los | Tel | 6 weeks |
| Zhu 2015 [91] | China | 60 | 64.1 | 41.7 | NA | NA | Los | Val | 4 weeks |
| Chen 2015 [92] | China | 70 | 62.9 | 57.1 | 540 | NA | Los | Ena | 4 weeks |
| Li 2016 [93] | China | 76 | 74.5 | 55.3 | 550 | Male: > 416 μmol/L; female: > 357 μmol/L | Irb + Adp | Irb | 24 weeks |
| Liu 2016 [94] | China | 95 | 51.8 | 56.8 | 440 | Male: > 420 μmol/L; female: > 360 μmol/L | Val | Fos | 8 weeks |
| Yang 2016 [95] | China | 100 | 60-78 | 54.0 | 550 | NA | Irb + Adp | Irb | 24 weeks |
| Yue 2016 [96] | China | 120 | 53.1 | 56.7 | 560 | Male: > 420 μmol/L; female: > 360 μmol/L | Los + Ndp | Ndp | 8 weeks |
| Yang 2016 [97] | China | 80 | 67.4 | 62.5 | 490 | > 416 μmol/L | Los | Can | 8 weeks |
| Mi 2016 [98] | China | 90 | 61.5 | 51.1 | 540 | > 416 μmol/L | Val + Adp | Adp | 12 weeks |
| He 2016 [99] | China | 98 | 65.8 | 58.2 | 550 | Male: > 416 μmol/L; female: > 357 μmol/L | Irb + Adp | Irb | 24 weeks |
| Lai 2016 [100] | China | 72 | 60.9 | 59.7 | 430 | NA | Los | Irb | 12 weeks |
| Li 2016 [101] | China | 150 | 60.3 | 64.7 | 520 | NA | Los + HCTZ | Ndp | 8 weeks |
| Xu 2016 [102] | China | 136 | 74.0 | 55.1 | 550 | Male: > 416 μmol/L; female: > 357 μmol/L | Irb + Adp | Irb | 24 weeks |
| Huang 2016 [103] | China | 100 | 68.8 | 55.0 | 550 | NA | Irb + Adp | Irb | 24 weeks |
| Zhang 2016 [104] | China | 104 | 64.6 | 55.8 | 450 | > 416 μmol/L | Irb + Adp | Adp | 12 weeks |
| Han 2016 [105] | China | 70 | 68.3 | 58.6 | 480 | Male: > 420 μmol/L; female: > 360 μmol/L | Los | Val | 8 weeks |
| Zhao 2017 [106] | China | 110 | 74.7 | 50.9 | 510 | > 390 μmol/L | Irb + Adp | Irb | 24 weeks |
| Wang 2017 [107] | China | 100 | 73.9 | 58.0 | 560 | NA | Irb + Adp | Irb | 24 weeks |
| Huang 2017 [108] | China | 200 | 67.9 | 55.0 | 550 | NA | Irb + Adp | Irb | 24 weeks |
| Wu 2017 [109] | China | 80 | 45.8 | 56.3 | 470 | Male: > 390 μmol/L; female: > 350 μmol/L | Los | Val | 4 weeks |
| Li 2017 [110] | China | 72 | 57.2 | 51.4 | 450 | > 416 μmol/L | Los | Irb | 24 weeks |
| Wang 2017 [111] | China | 68 | 63.6 | 66.2 | 480 | Male: > 420 μmol/L; female: > 360 μmol/L | Los | Val | 8 weeks |
| Mei 2017 [112] | China | 126 | 69.3 | 56.3 | 560 | > 416 μmol/L | Irb + Adp | Irb | 24 weeks |
| Zhu 2017 [113] | China | 86 | 69.6 | 65.1 | 490 | > 416 μmol/L | Los | Can | 8 weeks |
| Zhu 2017 [114] | China | 100 | 66.0 | 58.0 | 550 | Male: > 420 μmol/L; female: > 360 μmol/L | Irb + Adp | Adp | 24 weeks |
| Wu 2017 [115] | China | 62 | 65.9 | 62.9 | NA | NA | Los + Adp | Adp | 8 weeks |
| Shen 2017 [116] | China | 80 | 61.3 | 56.3 | 480 | Male: > 420 μmol/L; female: > 380 μmol/L | Los | Adp | 4 weeks |
| Han 2017 [117] | China | 114 | 72.4 | 63.2 | 500 | NA | Irb + Ndp | Ndp | 2 weeks |
| Jing 2017 [118] | China | 72 | 68.2 | 51.4 | 520 | NA | Los | Val | 8 weeks |
| Wang 2017 [119] | China | 68 | 66.0 | 54.4 | 480 | > 360 μmol/L | Los | Val | 8 weeks |
| Zhou 2017 [120] | China | 86 | 62.1 | 60.5 | 480 | Male: > 420 μmol/L; female: > 360 μmol/L | Los | Val | 8 weeks |
| Gao 2017 [121] | China | 106 | 66.8 | 60.4 | 490 | NA | Los | Tel | 8 weeks |
| Lu 2017 [122] | China | 80 | 62.1 | 68.8 | 490 | NA | Los | Val | 8 weeks |
| Wang 2018 [123] | China | 98 | 68.7 | 55.1 | 550 | Male: > 420 μmol/L; female: > 360 μmol/L | Irb + Adp | Irb | 24 weeks |
| Wang 2018 [124] | China | 108 | 54.2 | 58.3 | 520 | > 420 μmol/L | Los + Adp | Los | 8 weeks |
| Du 2018 [125] | China | 80 | 54.5 | 57.5 | 440 | NA | Los | Fdp | 6 weeks |
| Chen 2018 [126] | China | 90 | 64.2 | 58.9 | NA | NA | Irb + Adp | Adp | 24 weeks |
| Chen 2018 [127] | China | 280 | 32-85 | 57.9 | 390 | Male: > 420 μmol/L; female: > 357 μmol/L | Los | Adp | 8 weeks |
| Wu 2018 [128] | China | 64 | 66.4 | 60.9 | 440 | NA | Los | Ena | 6 weeks |
| Huang 2018 [129] | China | 116 | 72.4 | 55.2 | 560 | NA | Irb + Adp | Adp | 24 weeks |
| Chen 2018 [130] | China | 78 | 59.2 | 75.6 | 480 | NA | Los | Tel | 8 weeks |
| Li 2018 [131] | China | 105 | NA | NA | 560 | Male: > 416 μmol/L; female: > 357 μmol/L | Irb + Adp | Irb, Adp | 24 weeks |
| Sun 2018 [132] | China | 150 | 70.6 | 58.0 | 550 | NA | Irb + Adp | Adp | 24 weeks |
| Song 2019 [133] | China | 100 | 69.0 | 57.0 | 530 | Male: > 420 μmol/L; female: > 350 μmol/L | Los + Ndp | Ndp | 8 weeks |
| Wang 2019 [134] | China | 90 | 71.5 | 54.4 | 550 | > 416 μmol/L | Irb + Adp | Irb | 24 weeks |
| Gao 2019 [135] | China | 80 | 70.6 | 52.5 | 550 | Male: > 416 μmol/L; female: > 357 μmol/L | Irb + Adp | Irb | 24 weeks |
| Jiang 2019 [136] | China | 100 | 51.8 | 62.0 | 510 | Male: > 420 μmol/L; female: > 360 μmol/L | Los | All | 8 weeks |
| Zou 2019 [137] | China | 80 | 58.7 | 52.5 | 500 | NA | Los | Val | 12 weeks |
| Zhang 2019 [138] | China | 150 | 67.7 | 83.3 | 550 | NA | Irb + Adp | Irb | 24 weeks |
| Pei 2019 [139] | China | 84 | 70.6 | 56.0 | 510 | > 416 μmol/L | Los + Adp | Adp | 8 weeks |
| Zhang 2019 [140] | China | 64 | 64.1 | 54.7 | 560 | Male: > 420 μmol/L; female: > 360 μmol/L | Los + Adp | Adp | 12 weeks |
| Shen 2019 [141] | China | 74 | 66.5 | 56.8 | 440 | NA | Los | Can | 8 weeks |
| Su 2019 [142] | China | 80 | 65.5 | 53.8 | 480 | Male: > 420 μmol/L; female: > 360 μmol/L | Los | Val | 8 weeks |
| Li 2019 [143] | China | 82 | 60.5 | 56.1 | 480 | Male: > 420 μmol/L; female: > 360 μmol/L | Los | Val | 8 weeks |
| Wang 2019 [144] | China | 84 | 70.8 | 64.3 | 490 | Male: > 416 μmol/L; female: > 357 μmol/L | Irb + Adp | Adp | 12 weeks |
| Li 2019 [145] | China | 72 | 70.2 | 56.9 | 550 | > 416 μmol/L | Irb + Adp | Irb | 24 weeks |
| Ji 2019 [146] | China | 88 | 71.8 | 55.7 | 480 | Male: > 420 μmol/L; female: > 360 μmol/L | Los | Val | 8 weeks |
| Lin 2019 [147] | China | 50 | 58.6 | 74.0 | 480 | Male: > 420 μmol/L; female: > 360 μmol/L | Los | Tel | 8 weeks |
| Wang 2019 [148] | China | 110 | 58.0 | 57.3 | 550 | Male: > 420 μmol/L; female: > 357 μmol/L | Los + Adp | Los | 6 weeks |
| Xiong 2020 [149] | China | 120 | 60.1 | 51.7 | 420 | > 420 μmol/L | Los | Ben | 48 weeks |
| Xian 2020 [150] | China | 124 | 56.1 | 62.1 | 520 | > 420 μmol/L | Los + Adp | Los | 8 weeks |
| Li 2020 [151] | China | 60 | 66.5 | 63.3 | 430 | NA | Irb + Adp | Adp | 12 weeks |
| Sun 2020 [152] | China | 58 | 66.5 | 63.8 | 480 | NA | Los | Val | 8 weeks |
| Liu 2020 [153] | China | 100 | 58.8 | 49.0 | 420 | NA | Los + Adp | Los | 8 weeks |
| Zhou 2020 [154] | China | 102 | 58.9 | 51.0 | 470 | Male: > 420 μmol/L; female: > 340 μmol/L | Los + HCTZ | Ndp | 8 weeks |
| Tang 2020 [155] | China | 60 | 65.1 | 43.3 | 440 | Male: > 420 μmol/L; female: > 360 μmol/L | Val | All | 8 weeks |
| Tian 2020 [156] | China | 60 | 65.5 | 56.7 | 500 | Male: > 420 μmol/L; female: > 360 μmol/L | Los | Irb | 12 weeks |
| Liang 2020 [157] | China | 62 | 60.6 | 53.2 | NA | > 416 μmol/L | Los | Tel | 8 weeks |
| Fan 2020 [158] | China | 78 | 73.1 | 57.7 | 550 | Male: > 416 μmol/L; female: > 375 μmol/L | Irb + Adp | Irb | 24 weeks |
| Huang 2020 [159] | China | 150 | 73.1 | 55.3 | 550 | Male: > 420 μmol/L; female: > 360 μmol/L | Irb + Adp | Irb | 12 weeks |
| Zhang 2020 [160] | China | 84 | 65.5 | 58.3 | 550 | Male: > 416 μmol/L; female: > 357 μmol/L | Irb + Adp | Adp | 24 weeks |
| Shi 2021 [161] | China | 86 | 70.0 | 47.7 | 560 | NA | Irb + Adp | Irb | 24 weeks |
| Zhu 2021 [162] | China | 128 | 70.5 | 57.8 | 560 | Male: > 416 μmol/L; female: > 357 μmol/L | Irb + Adp | Irb | 24 weeks |
| Chang 2021 [163] | China | 100 | 63.5 | 55.0 | 490 | Male: > 420 μmol/L; female: > 360 μmol/L | Los | Val | 4 weeks |
| Xie 2021 [164] | China | 80 | 76.5 | 56.3 | 490 | > 416 μmol/L | Irb + Adp | Adp | 12 weeks |
| Wu 2021 [165] | China | 82 | 61.3 | 58.5 | 480 | > 444 μmol/L | Irb + Adp | Irb | 24 weeks |
| Ge 2021 [166] | China | 90 | 59.5 | 88.9 | 550 | > 416 μmol/L | Los + Adp | Los | 8 weeks |
| Li 2022 [167] | China | 240 | 75.7 | 57.9 | 420 | Male: > 390 μmol/L; female: > 310 μmol/L | Los | Irb | 12 weeks |
| Tan 2022 [168] | China | 100 | 65.2 | 52.0 | 490 | > 416 μmol/L | Val + Ndp | Ndp | 8 weeks |
| Wu 2022 [169] | China | 60 | 59.3 | 55.0 | 480 | NA | Los | Val | 8 weeks |
| Lin 2023 [170] | China | 100 | 66.9 | 60.0 | 580 | NA | Los + Adp | Adp | 8 weeks |
| Sheng 2023 [171] | China | 60 | 53.9 | 85.0 | 460 | 420-480 μmol/L | Los | All | 12 weeks |
| Sun 2023 [172] | China | 78 | 73.8 | 57.7 | 510 | > 416 μmol/L | Los + Adp | Adp | 8 weeks |

*Adp: amlodipine; All: Allisartan; Ben: Benazepril; Can: Candesartan; Cap: Captopril; Ena: Enalapril; Fdp: Felodipine; Fos: Fosinopril; HCTZ: Hydrochlorothiazide; Irb: Irbesartan; Lis: Lisinopril; Los: Losartan; NA: not available; Ndp: Nifedipine; Per: Perindopril; Tel: Telmisartan; Val: Valsartan

**Reference**

1. Wang H, Zhuo Y, Zheng B, et al. Comparison of efficacy of losartan potassium and fosinopril in the treatment of hypertension and hyperuricemia in elderly patients [J]. Foreign Medicine. Journal of Cardiovascular Diseases, 2000(05): 294-296.
2. Zhang Y, Liu F. Clinical observation of losartan in the treatment of hypertension combined with hyperuricemia [J]. Journal of Lingnan Cardiology, 2001(03):177-179.
3. Yang L, JIN X, GAO H. Clinical observation of valsartan in the treatment of elderly hypertension complicated with hyperuricemia [J]. Chinese Journal of Gerontology,2001(03):192-193.
4. Xu W, ZHANG S, XIAO J et al. Clinical observation of losartan in the treatment of hypertension complicated with hyperuricemia [J]. Foreign medicine. Journal of Cardiovascular Diseases,2001(01):41-43.
5. Ma C, WU X, Ding J. Clinical observation of losartan in the treatment of hypertension complicated with hyperuricemia [J]. Chinese Journal of Civil Medicine,2002(02):85-109.
6. Chen Y, Cao H. Clinical observation of ibesartan in the treatment of hypertension complicated with hyperuricemia [J]. Chinese Journal of Primary Medicine,2003(12):52.
7. Nie J, Chen H. Effect of losartan on hypertension complicated with hyperuricemia [J]. Journal of Cardiovascular Rehabilitation Medicine,2003(02): 170-172.
8. Xu G. Efficacy of losartan and felodipine in the treatment of hypertension combined with hyperuricemia in middle-aged and elderly patients [J]. Shanghai Journal of Preventive Medicine,2004(06):266-268.
9. Zhang X, Wang Z, Zhang H. Clinical study of losartan in the treatment of senile hypertension with hyperuricemia [J]. Journal of Xinjiang Medical University, 2005(09):832-834.
10. Zhang Y. Comparison of losartan and lisinopril in the treatment of hypertension combined with hyperuricemia [J]. Journal of the Fourth Military Medical University,2005(14):1292.
11. Dang A, Zhang Y, Liu G, Chen G, Song W, Wang B. Effects of losartan and irbesartan on serum uric acid in hypertensive patients with hyperuricaemia in Chinese population. J Hum Hypertens. 2006 Jan;20(1):45-50.
12. Zhang S, Huang L, Wang X. Clinical observation of losartan in the treatment of hypertension with hyperuricemia [J]. Zhejiang Preventive Medicine,2006(04): 77.
13. Zhu Z, Zhong D, Zheng W et al. Effect of losartan on the treatment of essential hypertension complicated with hyperuricemia [J]. Chinese Journal of Hypertension,2006(12):1015-1016.
14. Du S, Yue X, ZHANG Xiaojian et al. Clinical study of losartan in the treatment of essential hypertension complicated with hyperuricemia [J]. Zhongyuan Medical Journal,2006(21):26-27.
15. Zheng X, HAO Z, WANG Y. Clinical study of losartan in the treatment of hypertension with hyperuricemia [J]. Zhongyuan Medical Journal,2006(15):5-6.
16. Liu Y. Comparison of effects of losartan and amlodipine on blood pressure and blood uric acid in patients with hypertension [J]. Journal of Dalian Medical University,2006(03):222-223.
17. Li R, Li J. Clinical observation of losartan in the treatment of hypertension complicated with hyperuricemia [J]. Hainan Medicine,2006(06):1-3.
18. Fan G, Xu X. Comparative observation of the antihypertensive and uric acid effects of losartan and benazepril [J]. Shaanxi Medical Journal,2006(05):598-599.
19. Chen H, LI Y, ZHANG X, et al. Clinical observation of valsartan in the treatment of hypertension complicated with hyperuricemia [J]. Hebei Med, 2007(01):17-18.
20. Zhang Y. Clinical observation of losartan in the treatment of essential hypertension complicated with hyperuricemia [J]. Modern Distance Education of Chinese Medicine,2008(03):250.
21. Gu X, Liang Y. Losartan in the treatment of essential hypertension complicated with hyperuricemia [J]. Journal of Lingnan Cardiology,2008(05):353-354.
22. Zhao X, Guard C. Clinical analysis of irbesartan in the treatment of hypertension combined with hyperuricemia in middle-aged and elderly patients [J]. China Primary Health Care,2008(11):85-86.
23. Yuan HP. Effect of valsartan on uric acid metabolism in patients with hypertension complicated with hyperuricemia [J]. Modern Medicine and Health, 2008(18):2728-2729.
24. Qi L, Feng Y, ZHAO L. Clinical observation of antihypertensive and uric acid effects of losartan and enalapril [J]. Chinese Factory and Mining Medicine,2008(01):46.
25. Chen Z, Chen Y, Xi K, et al. Observation on the therapeutic effect of combined use of Valsartan and sustained-release Nifedipine in elderly patients with primary hypertension accompanied by hyperuricemia[J]. Hainan Medicine, 2008(02): 12-13.
26. Zeng H, Yao Z, Shi S et al. Clinical study of angiotensin Ⅱ in the treatment of 52 patients with hypertension complicated with hyperuricemia [J]. Chinese Modern Medical Doctor,2009,47(30):38+70
27. Yuan ZL. Clinical observation of losartan in the treatment of 55 cases of hypertension complicated with hyperuricemia [J]. Chinese Community Physician (Bimonthly Medical Specialty),2009,11(19):34.
28. Zhang Q, Yang J. Losartan combined with amlodipine in the treatment of 31 cases of hypertension complicated with hyperuricemia [J]. Shaanxi Medical Journal,2009,38(08):1058-1060.
29. Jiang L. Clinical observation of losartan in the treatment of mild and moderate essential hypertension with hyperuricemia [J]. Youjiang Med,2009,37(03):267-268.
30. Li Y. Comparison of efficacy of losartan potassium and enalapril in the treatment of hypertension complicated with hyperuricemia [J]. Journal of Clinical and Experimental Medicine,2009,8(03):80-81.
31. CAI Y, Tang Y. Comparison of efficacy of losartan and Telmisartan in the treatment of hypertension with hyperuricemia [J]. Current Medicine,2009, 15(09): 129-130.
32. Liao J, Lin J. Clinical observation on the antihypertensive and uric acid effects of losartan and fosinopril [J]. Sichuan Medical Journal,2009,30(02): 234-235.
33. Cheng H, Lu H. Clinical observation of telmisartan in the treatment of hypertension complicated with hyperuricemia [J]. Journal of Nantong University (Medical Edition),2010,30(06):482+484.
34. Tian M, Zhang J. Comparison of the effects of losartan and irbesartan on essential hypertension complicated with hyperuricemia [J]. International Medical Journal,2010,29(35):99-100.
35. Wang J. Efficacy of valsartan and levamlodipine in the treatment of essential hypertension and influence on blood uric acid [J]. Medical Information (Mid-week), 2010,5(09):2316-2317.
36. Gong Q.Clinical observation of the antihypertensive and uric acid effects of losartan and Lisinopril [J]. Chinese Modern Medical Doctor,2010,48(12):106-107.
37. Wang Q, HUANG X, ZHANG J. Effects of irbesartan on blood uric acid metabolism and left ventricular function in hypertensive patients with hyperuricemia [J]. Chinese Medicine,2010,23(02):136-139.
38. Zhang Q. Clinical observation on the antihypertensive and uric acid effects of losartan and fosinopril [J]. Chinese Community Physician (Medical Profession), 2010,12(08):31.
39. Xie YG. Losartan in the treatment of essential hypertension complicated with hyperuricemia [J]. Medical Theory & Practice,2010,23(02):157-158.
40. Zhang T, Zhang C. Irbesartan combined with amlodipine besylate in the treatment of elderly diabetic patients with hypertension and hyperuricemia [J]. Chinese Journal of Clinical Medicine,2011,39(12):25-27.
41. Li Yong, Qian Xiaoxia, Wu Jianbo, et al. Efficacy of losartan in the treatment of senile hypertension with hyperuricemia [J]. People's Military Medicine,2011,54(11):973-974.
42. Wu X.Clinical observation of losartan in the treatment of 60 cases of hypertension with hyperuricemia [J]. Journal of Internal Medicine,2011,6(03):222-223.
43. He Lei, Zheng Yonghong. Comparison of efficacy of valsartan and enalapril in the treatment of hypertension complicated with hyperuricemia [J]. Inner Mongolia Traditional Chinese Medicine,2011,30(11):12.
44. Zeng Zhi, LU Dongfeng, ZENG Zhaohua et al. Effects of losartan potassium and irbesartan on blood pressure and insulin sensitivity in patients with hypertension and hyperuricemia [J]. Chinese Journal of Modern Pharmaceutical Application,2011,5(02):8-10.
45. Xia Weibing, Pan Yaosheng, Lv Liyou. Comparison of efficacy of losartan and fosinopril in the treatment of essential hypertension complicated with hyperuricemia [J]. Chinese Community Physician (Medical Specialty),2011,13(09):111-112.
46. Wu S, Bao Z. Comparison of efficacy of losartan and valsartan in the treatment of essential hypertension complicated with hyperuricemia [J]. Chinese Journal of Modern Applied Pharmacy, 2012, 29(12):1137-1139.
47. Tian Ming, HAO Qiyun, LU Ziming et al. Comparative study of efficacy of losartan and fosinopril in patients with hypertensive left ventricular hypertrophy complicated with hyperuricemia [J]. Chinese Modern Medical Journal,2012,50(35):74-76.
48. Lu R. Efficacy of losartan combined with amlodipine in the treatment of hypertension complicated with hyperuricemia [J]. Jilin Medicine,2012, 33(23):5015.
49. Li L S. Comparative observation of losartan and Perindopril in the treatment of hypertension combined with hyperuricemia [J]. Chinese Journal of Modern Pharmaceutical Application,2012,6(15):64-65.
50. Shao H. Study on the therapeutic effect of irbesartan on hypertensive patients with hyperuricemia [J]. Chinese Journal of Primary Health Care,2012,26(08): 88-89.
51. Zhao H, Chen L, Zhou X. Efficacy of losartan and Telmisartan in the treatment of hypertension with hyperuricemia [J]. Journal of Qiqihar Medical College, 2013,34(16):2403-2404.
52. Wang Y, Liu Y. Efficacy of losartan potassium combined with amlodipine in the treatment of essential hypertension complicated with hyperuricemia [J]. Chinese Modern Medical Journal,2013,51(20):57-58.
53. Tang B. Efficacy of losartan in the treatment of elderly patients with hypertension complicated with hyperuricemia [J]. Chinese Journal of Practical Medicine,2013,8(17):38-39.
54. Zhang W, Xu Y. Efficacy of losartan potassium tablets in the treatment of community hypertension complicated with hyperuricemia [J]. Chinese Community Physicians (Medical Specialties),2013,15(10):57.
55. Xu L. Clinical observation of irbesartan combined with hydrochlorothiazide in the treatment of essential hypertension complicated with hyperuricemia [J]. Chinese Journal of Health and Nutrition,2013,23(02):314.
56. Song H. Efficacy of losartan potassium and felodipine in the treatment of elderly hypertension complicated with hyperuricemia [J]. China Prescription Drugs, 2014,12(11):39-40.
57. Zhang X, Ma L, Zheng L. Effect of Irbesartan combined with amlodipine besylate on elderly patients with diabetic hypertension complicated with hyperuricemia [J]. Chinese Journal of Biochemical Drugs,2014,34(08): 157-158+161.
58. Wang M, Sun X. Clinical effect of amlodipine besylate combined with irbesartan in the treatment of hypertension complicated with hyperuricemia [J]. Journal of Nantong University (Medical Science Edition),2014,34(06):559-560.
59. Fang F.Curative effect of irbesartan on hypertension complicated with hyperuricemia [J]. International Medical Journal,2014,33(25):127-128.
60. Zhong L, Xiong S. Efficacy of losartan potassium combined with amlodipine in the treatment of essential hypertension complicated with hyperuricemia [J]. South China Journal of Defense Medicine,2014,28(08):772-773.
61. Wang B. Efficacy of valsartan in the treatment of hypertension complicated with hyperuricemia [J]. Journal of Industrial Medicine,2014,27(04):872-873.
62. Yao X. Effects of losartan on blood pressure and blood uric acid level in patients with essential hypertension complicated with hyperuricemia [J]. Chinese Journal of Geriatric Medicine,2014,12(03):53-55.
63. Yang L. Efficacy of losartan potassium tablets in the treatment of hypertension combined with hyperuricemia [J]. Journal of Clinical Rational Use of Medicine,2014,7(18):29-30.
64. Chen C.Curative effect of irbesartan combined with Lodipine besylate in the treatment of elderly patients with diabetic hypertension complicated with hyperuricemia [J]. Chinese Journal of Gerontology,2014,34(11):2946-2948.
65. Meng W. Clinical efficacy of losartan in the treatment of hypertension complicated with hyperuricemia [J]. Chinese Medical Guide,2014,12(07):78-79.
66. Tang W. Effect of losartan on patients with hypertension and hyperuricemia [J]. Contemporary Medicine,2014,20(07):135.
67. Fang Z. Clinical efficacy of losartan in the treatment of hypertension combined with hyperuricemia [J]. Chinese Medical Guide,2014,12(03):122-123.
68. Yan Y, Song J. Clinical observation of Irbesartan combined with Lodipine besylate in the treatment of elderly patients with diabetes mellitus complicated with hypertension and hyperuricemia [J]. Journal of Youjiang Medical College for Nationalities,2015,37(05):692-693.
69. Wei X. Curative effect of irbesartan combined with amlodipine besylate on elderly diabetic hypertension complicated with hyperuricemia [J]. Chinese Community Physician,2015,31(31):18+20.
70. Cui S. Curative effect of Irbesartan combined with amlodipine besylate in the treatment of elderly diabetic hypertension complicated with hyperuricemia [J]. Journal of Cardio-Cerebrovascular Diseases of Integrated Chinese and Western Medicine,2015,13(18):2084-2086.
71. Zhang B. Effects of losartan on blood pressure and uric acid metabolism in patients with hypertension complicated with hyperuricemia [J]. China Pharmaceutical Industry,2015,24(24):133-134.
72. Sun N. Curative effect of irbesartan combined with amlodipine in the treatment of elderly diabetes mellitus with hypertension and high uric acid [J]. Journal of Practical Diabetes,2015,11(06):25-26.
73. Lu P, Xu Y, Mei Z. Clinical value of irbesartan combined with amlodipine besylate in the treatment of elderly patients with diabetic hypertension complicated with hyperuricemia [J]. Current Medicine,2015,21(29):131-132.
74. Zhang C. Curative effect of irbesartan plus amlodipine besylate in the treatment of elderly diabetic hypertension complicated with hyperuricemia [J]. Modern Diagnosis and Therapy,2015,26(19):4374-4376.
75. Wang P. Losartan treating 139 cases of essential hypertension with high uric acid [J]. China Pharmaceutical Industry,2015,24(17):122-123.
76. Yuan F, Wang L, Gao J et al. Clinical study of losartan potassium in the treatment of essential hypertension with hyperuricemia [J]. Chinese Journal of Clinical Pharmacology,2015,31(16):1566-1568.
77. Liao D. Curative effect of irbesartan combined with Lodipine besylate in the treatment of elderly diabetic hypertension complicated with hyperuricemia [J]. Chinese and Foreign Medical Research,2015,13(24):32-33.
78. Shi F. Curative effect of Irbesartan combined with Lodipine benzenesulfonate in the treatment of elderly diabetic hypertension complicated with hyperuricemia [J]. Journal of Cardiovascular Diseases of Integrated Chinese and Western Medicine,2015,3(23):114-115.
79. Li J. Efficacy and safety evaluation of combined drug therapy for essential hypertension complicated with hyperuricemia [J]. Chinese Journal of Health Standards,2015,6(15):122-123.
80. Ji C. Clinical effect analysis of irbesartan combined with amlodipine besylate in elderly patients with diabetic hypertension complicated with hyperuricemia [J]. Cardiovascular disease Prevention and Treatment Knowledge (Academic Edition),2015(12):23-24.
81. Zuo N, Wang C. Efficacy of losartan potassium and enalapril in the treatment of elderly essential hypertension complicated with hyperuricemia [J]. Chinese Journal of Geriatric Medicine,2015,13(03):43+46.
82. Zhong Y. Curative effect of Irbesartan combined with Lodipine besylate in the treatment of 40 elderly patients with diabetic hypertension complicated with hyperuricemia [J]. Clinical Research of Chinese Medicine,2015,7(36):118.
83. Dong Y. Observation of irbesartan combined with amlodipine besylate in the treatment of elderly diabetic hypertension complicated with hyperuricemia [J]. Chinese Journal of Practical Medicine,2015,10(17):182-183.
84. Wu D. Curative effect of Irbesartan combined with amlodipine besylate in the treatment of elderly diabetic hypertension complicated with hyperuricemia [J]. Journal of Practical Clinical Medicine,2015,16(05):13-14.
85. Jin L.Comparison of efficacy of losartan and Telmisartan in the treatment of hypertension with hyperuricemia [J]. Chinese Journal of Medicine,2015,27(05):120-121.
86. Li C, Niu Y. Efficacy of irbesartan combined with Lodipine besylate in the treatment of elderly diabetic hypertension complicated with hyperuricemia [J]. Chinese Modern Pharmaceutical Application,2015,9(01): 98-99.
87. Li H. Efficacy of Valsartan combined with Nifedipine air release tablets in the treatment of hypertension complicated with hyperuricemia [J]. Chinese and Foreign Medical Journal,2015,34(04):95-97.
88. Zhong W. Efficacy of Irbesartan combined with Lodipine besylate in the treatment of elderly diabetic hypertension complicated with hyperuricemia [J]. New World of Diabetes Mellitus,2015(04):27.
89. Zhang J.Clinical observation of valsartan in the treatment of essential hypertension with hyperuricemia [J]. Chinese Journal of Medical Sciences,2015, 5(04):52-53+62.
90. Han F. Effects of Telmisartan and losartan on renal hypertension complicated with hyperuricemia [J]. Chinese Contemporary Medicine,2015,22(06): 140-141+144.
91. Zhu H. Effect of losartan potassium in the treatment of community hypertension patients with hyperuricemia [J]. Primary Medicine Forum,2015, 19(11):1464-1465.
92. Chen H. Comparison of efficacy of losartan and enalapril in the treatment of hypertension combined with hyperuricemia [J]. Straits Pharmacy,2015,27(04): 144-145.
93. Li W. The effect of irbesartan combined with lodipine benzenesulfonate in the treatment of elderly diabetes mellitus with hypertension and hyperuricemia [J]. North China Pharmacy,2016,13(01):56-57.
94. Liu Q, You D, Liang Y. Effect of valsartan and fosinopril in the treatment of elderly essential hypertension complicated with hyperuricemia [J]. North China Pharmacy,2016,13(01):7-8.
95. Yang Y, Jiang S, Wu W. Effect of Irbesartan combined with amlodipine besylate in the treatment of elderly patients with diabetic hypertension complicated with hyperuricemia [J]. Chinese Rural Health,2016(02):7.
96. Yue Y. Curative effect of Losartan potassium combined with Nifedipine in the treatment of hypertension with hyperuricemia [J]. Chinese Hospital Drug Evaluation and Analysis,2016,16(01):18-20.
97. Yang P, WANG L, YUAN F. Clinical efficacy and safety evaluation of losartan potassium in the treatment of elderly essential hypertension with hyperuricemia [J]. Chinese Journal of Clinical Pharmacology,2016,32(04):300-302.
98. Mi Z. Effect of valsartan combined with amlodipine besylate on patients with essential hypertension complicated with hyperuricemia [J]. Medical Theory and Practice,2016,29(06):745-746.
99. He D, ZHAO X, Deng X. Therapeutic effect of Irbesartan combined with Lodipine besylate in the treatment of elderly diabetic hypertension complicated with hyperuricemia [J]. Shenzhen Journal of Integrated Chinese and Western Medicine,2016,26(07):6-7.
100. Lai X. Effects of losartan on serum uric acid and urinary protein in hypertensive patients with hyperuricemia [J]. Modern Hospital,2016, 16(04): 509-510.
101. Li Y, QIN F, Yi H. Effect of losartan combined with hydrochlorothiazide on blood pressure and blood uric acid in hypertensive patients with hyperuricemia [J]. Chinese Rural Health,2016(08):12.
102. Xu X, Qiu Q, Gao X, et al. Effect of Irbesartan combined with amlodipine besylate on elderly patients with diabetic hypertension complicated with hyperuricemia [J]. Hebei Medical Journal,2016,22(05):764-766.
103. Huang Y. Efficacy of Irbesartan combined with amlodipine besylate in the treatment of elderly diabetic hypertension complicated with hyperuricemia [J]. Straits Pharmacy,2016,28(06):158-159.
104. Zhang S. Efficacy of combination of irbesartan and amlodipine in the treatment of hypertensive patients with hyperuricemia [J]. Journal of Contemporary Medicine,2016,14(15):113-115.
105. Han J, Li J, Luo P, et al. Comparison of clinical effect of losartan and valsartan in the treatment of hypertension combined with hyperuricemia [J]. Chongqing Medical College,2016,45(26):3707-3708.
106. Zhao G. Effect of Irbesartan combined with amlodipine besylate in the treatment of elderly diabetic hypertension complicated with hyperuricemia [J]. Journal of Clinical Medicine,2017,37(11):124-126.
107. Wang Y. Clinical effect of irbesartan combined with levamlodipine in the treatment of diabetes mellitus with hyperuricemia [J]. Journal of Clinical Medicine Literature Electronic,2017,4(91):17953-17954.
108. Huang T.Clinical analysis of amlodipine besylate combined with irbesartan in the treatment of elderly diabetic hypertension complicated with hyperuricemia [J]. Inner Mongolia Medical Journal,2017,49(10):1244-1245.
109. Wu L, Jiang Y, Su H, et al. Effect of losartan on blood uric acid in patients with hypertension combined with hyperuricemia [J]. Journal of Cardio-Cerebrovascular Diseases of Integrated Chinese and Western Medicine, 2017,15(14):1758-1760.
110. Li A, Liu Y, Zhu W. Effect of losartan on serum uric acid in hypertensive patients with hyperuricemia [J]. Chinese Journal of Chronic Medicine,2017, 18(06):627-629.
111. Wang Y. Comparison of clinical effects of losartan and valsartan in the treatment of hypertension combined with hyperuricemia [J]. Chinese Medical Guide,2017,15(15):131-132.
112. Mei C. Effect of amlodipine besylate combined with irbesartan in the treatment of elderly patients with essential hypertension complicated with hyperuricemia [J]. Chinese Journal of Rural Medicine,2017,24(10):12-13.
113. Zhu Y. Clinical efficacy and safety evaluation of losartan potassium in the treatment of elderly essential hypertension with hyperuricemia [J]. Bipodia and Health Care,2017,26(09):125-126.
114. Zhu X, LIU Y, Han S, et al. Clinical observation of irbesartan in the treatment of hypertension, hyperuricemia and left ventricular hypertrophy [J]. Chinese Journal of Modern Pharmaceutical Application,2017,11(08):14-16.
115. Wu C. Clinical effect of losartan potassium combined with levamlodipine in the treatment of hypertension with hyperuricemia [J]. Chinese Community Physician,2017,33(10):37+39.
116. Shen Q, Gu J. Curative effect of losartan potassium in the treatment of community hypertension complicated with hyperuricemia [J]. Journal of Clinical Rational Drug Use,2017,10(07):50-51.
117. Han X, Zhang X. Effect of irbesartan in the treatment of hypertension patients with hyperuricemia and type 2 diabetes [J]. Journal of Contemporary Medicine, 2017,15(04):89-90.
118. Jing F. Comparison of clinical efficacy of losartan and valsartan in the treatment of hypertension combined with hyperuricemia [J]. Journal of Clinical Medical Literature Electronic,2017,4(07):1320-1321.
119. Wang Y. Comparison of clinical effects of losartan and valsartan in the treatment of hypertension combined with hyperuricemia [J]. Chinese Journal of Chronic Medicine,2017,18(01):55-56.
120. Zhou J, Gu J. Comparison of clinical effects of losartan and valsartan in the treatment of hypertension combined with hyperuricemia [J]. Journal of Clinical Medical Literature Electronic,2017,4(04):762.
121. Gao Y. Comparative study on the efficacy of losartan and Telmisartan in the treatment of hypertension with hyperuricemia [J]. Journal of Clinical Medical Literature Electronic,2017,4(02):350.
122. Lu Y. Clinical observation of losartan potassium in the treatment of essential hypertension complicated with hyperuricemia [J]. World Latest Medical Information Abstracts,2017,17(01):86.
123. Wang J. Effect of irbesartan combined with amlodipine besylate on elderly patients with diabetes mellitus combined with hypertension and hyperuricemia [J]. Journal of Henan Medical College,2018,30(06):561-563.
124. Wang Y. Losartan potassium combined with amlodipine in the treatment of essential hypertension complicated with hyperuricemia [J]. Northern Pharmacology,2018,15(12):6-7.
125. Du L. Effect of losartan potassium on blood pressure and uric acid in patients with essential hypertension complicated with hyperuricemia [J]. Chinese Medical Guide,2018,16(28):73.
126. Chen Y. Clinical observation of amlodipine combined with irbesartan in the treatment of hypertension with hyperuricemia and left ventricular hypertrophy [J].Infection International(Electronic Edition),2018,7(03):83-84.
127. Chen L, Han L, Luo J. Effect of amlodipine besylate and losartan potassium on uric acid level in patients with essential hypertension complicated with hyperuricemia [J]. Chinese Modern Pharmaceutical Application,2018,12(15): 137-138.
128. Wu X. Evaluation of losartan potassium tablets in the treatment of hypertension combined with hyperuricemia [J]. Chinese Journal of Chronic Medicine,2018,19(07):947-949.
129. Huang Y. Efficacy of Irbesartan combined with amlodipine besylate in the treatment of elderly patients with diabetic hypertension complicated with hyperuricemia [J]. Journal of Modern Medicine and Health Research,2018,2(09):51.
130. Chen Z. Clinical effect of losartan and Telmisartan in the treatment of hypertension with hyperuricemia [J]. Chinese Health Standard Management, 2018,9(08):84-85.
131. Li L. Clinical evaluation of irbesartan combined with amlodipine besylate in the treatment of elderly diabetes mellitus with hypertension and hyperuricemia [J]. Smart Health,2018,4(10):69-71.
132. Sun X. Clinical effect of drug combination on elderly diabetic hypertension complicated with hyperuricemia [J]. Journal of Practical Clinical Medicine,2018,22(01):133-135.
133. Song C. Effect of Losartan potassium tablets combined with Nifedipine sustained-release tablets in the treatment of hypertension complicated with hyperuricemia [J]. Chinese Journal of Medicine,2019,17(32):101.
134. Wang X. Clinical effect of amlodipine besylate combined with irbesartan in the treatment of elderly diabetic hypertension complicated with hyperuricemia [J]. Henan Medical Research,2019,28(21):3926-3927.
135. Gao X, WEI B. Curative effect of irbesartan combined with amlodipine besylate on elderly patients with diabetic hypertension complicated with hyperuricemia [J]. Chinese Journal of Geriatric Medicine,2019,17(05):63-64.
136. Jiang L, Du G, Chen R, et al. Effect of alisartan in the treatment of mild to moderate hypertension combined with hyperuricemia [J]. Journal of Lingnan Emergency Medicine,2019,24(05):485-486.
137. Zou W. The clinical value of losartan in the treatment of hypertension complicated with hyperuricemia [J]. Northern Pharmacy,2019,16(10):92-93.
138. Zhang L, Tang Y, Wang L. Clinical effect of irbesartan combined with amlodipine besylate in the treatment of elderly diabetic hypertension complicated with hyperuricemia [J]. Chinese Medical Guide,2019,17(26):20-21.
139. Bae Y, Han C. Efficacy of losartan potassium tablets in the treatment of elderly patients with essential hypertension and hyperuricemia [J]. Chinese Women's Health Research,2019(17):109-110.
140. Zhang L. Clinical efficacy and safety of amlodipine besylate combined with losartan potassium in the treatment of essential hypertension complicated with hyperuricemia [J]. Famous Medicine,2019(08):263.
141. Shen X, Fan W, Fu J. Clinical effect of losartan potassium on elderly patients with essential hypertension complicated with hyperuricemia [J]. Cardiovascular disease Prevention and Treatment Knowledge (Academic Edition), 2019,9(21):3-5.
142. Su X. Clinical efficacy of losartan and valsartan in the treatment of hypertension complicated with hyperuricemia [J]. Chinese Journal of Modern Pharmaceutical Application,2019,13(14):99-100.
143. Li X. Comparison of efficacy and pharmacoeconomics of losartan and valsartan in the treatment of hypertension combined with hyperuricemia [J]. Journal of Chronic Medicine,2019,20(06):928-930.
144. Wang S, Li Y. Effect of amlodipine and irbesartan in the treatment of elderly diabetic hypertension complicated with hyperuricemia [J]. Chinese Journal of Medical Sciences,2019,9(06):69-71.
145. Li Y. Amlodipine and irbesartan combined treatment of 36 elderly patients with diabetic hypertension complicated with hyperuricemia [J]. Journal of Mathematical Medicine and Pharmacology,2019,32(03):422-423.
146. Ji J. Clinical effect of losartan and valsartan in the treatment of hypertension combined with hyperuricemia [J]. Clinical Medicine Research and Practice,2019,4(07):31-32.
147. Lin Q, Yu B, Su D, et al. Clinical effect of Losartan and Telmisartan in the treatment of hypertension with hyperuricemia [J]. Guizhou Medicine,2019,43(02): 271-274.
148. Wang J. Comparison of efficacy of amlodipine besylate and losartan potassium in the treatment of EH complicated with HUA [J]. Clinical Journal of Integrated Chinese and Western Medicine,2019,19(01):115-116.
149. Xiong X, Zhang X. Study of losartan potassium in the treatment of essential hypertension with left ventricular hypertrophy and hyperuricemia [J]. Jiangxi Medicine, 2019,55(11):1655-1656.
150. Yun H, Liu X. Analysis of serum uric acid, urinary microprotein and arteriosclerosis index of amlodipine combined with losartan potassium in the treatment of hypertension complicated with hyperuricemia [J]. Journal of Cardio-Cerebrovascular Diseases of Integrated Chinese and Western Medicine,20,18(15):2495-2497.
151. Li D. Clinical effect of irbesartan combined with amlodipine in the treatment of diabetic hypertension complicated with hyperuricemia [J]. Journal of Clinical Rational Drug Use, 2019,13(19):56-58.
152. Sun Y. Therapeutic effect of losartan potassium in the treatment of community hypertension complicated with hyperuricemia [J]. Chinese Community Physician,20,36(17):64-65.
153. Liu J, Liu W. To observe the clinical efficacy and safety of amlodipine besylate combined with losartan potassium in the treatment of essential hypertension complicated with hyperuricemia [J]. Health for All,2020(09): 281-282.
154. Zhou K, WANG Y, WANG C. Effect of losartan combined with hydrochlorothiazide on patients with hypertension and hyperuricemia [J]. Chinese Journal of Practical Medicine, 2019,15(07):150-152.
155. Tang Y, Yang D, YI D. Effect of alisartan and valsartan in the treatment of hypertension complicated with hyperuricemia [J]. Journal of Hunan Normal University (Medical Edition),20,17(01):40-42.
156. Tian W. Clinical effect of losartan and irbesartan in the treatment of elderly essential hypertension with hyperuricemia [J]. Jilin Medical Journal, 2019,41(02):341-342.
157. Liang L, Wang J, Li B, et al. Comparative analysis of losartan and telmisartan in the treatment of hypertension with hyperuricemia [J]. Capital Food and Medicine, 2019,27(03):61-62.
158. Fan J. Clinical effect of irbesartan combined with amlodipine besylate in the treatment of elderly patients with diabetic hypertension complicated with hyperuricemia [J]. Chinese Community Physician, 2019,36(03):67-68.
159. Huang W. Curative effect of irbesartan + amlodipine besylate in the treatment of elderly diabetes mellitus with hypertension and hyperuricemia [J]. Chinese Journal of Practical Medicine, 2019,15(02):117-119.
160. Zhang J.Curative effect of irbesartan combined with amlodipine besylate in the treatment of diabetes mellitus with hypertension and hyperuricemia [J]. Journal of Mathematical Medicine and Pharmacology, 2019,33(01):110-111.
161. Shi J. Clinical value of irbesartan combined with amlodipine besylate in the treatment of elderly patients with diabetic hypertension complicated with hyperuricemia [J]. Chinese Community Physician, 21,37(27):35-36.
162. Zhu B. Clinical effect of drug combination on elderly diabetic hypertension complicated with hyperuricemia [J]. Journal of Practical Medical Technology, 201,28(03):388-390.
163. Chang S, HOU M, Xu H. Comparative study of clinical efficacy of losartan and valsartan in the treatment of hypertension complicated with hyperuricemia [J]. Journal of Clinical Rational Drug Use, 21,14(06):13-15.
164. Xie Q, Yang J. Effect of oral administration of amlodipine besylate and irbesartan on blood pressure and uric acid level in elderly patients with diabetes mellitus complicated with hypertension and hyperuricemia [J]. Chinese Community Physician, 201,37(06):70-71.
165. Wu C. Efficacy and adverse reactions of irbesartan and amlodipine besylate in the treatment of hypertension with hyperuricemia [J]. Journal of Northern Medicine, 2019,18(02):137-138.
166. Ge X. Effect of amlodipine besylate on blood pressure and blood uric acid in patients with essential hypertension complicated with hyperuricemia [J]. Journal of Modern Medicine and Health Research, 21,5(02):123-124.
167. Li L. Clinical treatment and effect observation of elderly patients with essential hypertension complicated with hyperuricemia [J]. Wisdom and Health, 2012,8(19):36-39.
168. Tan Z. Clinical study of valsartan combined with Nifedipine in the treatment of hypertension complicated with hyperuricemia [J]. Electronic Journal of Modern Medicine and Health Research,202,6(08):63-65.
169. Wu L. Comparative study on the clinical effect of losartan and valsartan in the treatment of hypertension combined with hyperuricemia [J]. Medical Diet and Health, 2012,20(07):71-73+76.
170. Lin X, Yang Q, Zheng F. Clinical efficacy of losartan potassium combined with amlodipine in the treatment of elderly patients with essential hypertension complicated with hyperuricemia [J]. Clinical Rational Use of drugs, 2019,16(24): 5-8.
171. Senda. Effect of alisartan ester and losartan potassium on hypertension with asymptomatic hyperuricemia [J]. Chinese Community Physician, 2019,39(19):24-26.
172. Sun J. Efficacy of losartan potassium tablets in the treatment of elderly patients with essential hypertension and hyperuricemia [J]. Chinese Journal of Modern Pharmaceutical Application, 2019,17(04):119-121.
