# Supplementary material for: Antihypertensive drugs for hyperuricemia in patients with hypertension: a systematic review and network meta-analysis of Chinese trials
Source: BMC Cardiovasc Disord. 2025 Dec 2;25:856. doi: 10.1186/s12872-025-05339-7 (PMC12673777; doi:10.1186/s12872-025-05339-7)
Supplement: Supplementary file 3 — Supplementary Material 3. [file 12872_2025_5339_MOESM3_ESM.docx]

Table S1. The methodological quality assessment of included trials

| Study | D1 | D2 | D3 | D4 | D5 |
| --- | --- | --- | --- | --- | --- |
| Wang 2000 [1] | Some concerns | Some concerns | Low risk | Some concerns | High risk |
| Zhang 2001 [2] | Some concerns | Some concerns | Low risk | Some concerns | High risk |
| Yang 2001 [3] | Some concerns | Some concerns | Low risk | Some concerns | High risk |
| Xu 2001 [4] | Some concerns | Some concerns | Low risk | Some concerns | High risk |
| Ma 2002 [5] | Some concerns | Some concerns | Low risk | Some concerns | High risk |
| Chen 2003 [6] | Some concerns | Some concerns | Low risk | Some concerns | High risk |
| Nie 2003 [7] | High risk | Some concerns | Low risk | Some concerns | High risk |
| Xu 2004 [8] | Some concerns | Some concerns | Low risk | Some concerns | High risk |
| Zhang 2005 [9] | Some concerns | Some concerns | Low risk | Some concerns | High risk |
| Zhang 2005 [10] | Some concerns | Some concerns | Low risk | Some concerns | High risk |
| Dang 2006 [11] | Low risk | Some concerns | Low risk | Some concerns | Low risk |
| Zhang 2006 [12] | Some concerns | Some concerns | Low risk | Some concerns | High risk |
| Zhu 2006 [13] | Low risk | Some concerns | Low risk | Some concerns | High risk |
| Du 2006 [14] | Some concerns | Some concerns | Low risk | Some concerns | High risk |
| Zheng 2006 [15] | High risk | Some concerns | Low risk | Some concerns | High risk |
| Liu 2006 [16] | Some concerns | Some concerns | Low risk | Some concerns | High risk |
| Li 2006 [17] | Some concerns | Some concerns | Low risk | Some concerns | High risk |
| Fan 2006 [18] | High risk | Some concerns | Low risk | Some concerns | High risk |
| Chen 2007 [19] | Some concerns | Some concerns | Low risk | Some concerns | High risk |
| Zhang 2008 [20] | Some concerns | Some concerns | Low risk | Some concerns | High risk |
| Gu 2008 [21] | Low risk | Some concerns | Low risk | Some concerns | High risk |
| Zhao 2008 [22] | Some concerns | Some concerns | Low risk | Some concerns | High risk |
| Yuan 2008 [23] | Low risk | Some concerns | Low risk | Low risk | High risk |
| Qi 2008 [24] | Some concerns | Some concerns | Low risk | Some concerns | High risk |
| Chen 2008 [25] | Some concerns | Some concerns | Low risk | Some concerns | High risk |
| Zeng 2009 [26] | Some concerns | Some concerns | Low risk | Some concerns | High risk |
| Yuan 2009 [27] | Some concerns | Some concerns | Low risk | Some concerns | High risk |
| Zhang 2009 [28] | Some concerns | Some concerns | Low risk | Some concerns | High risk |
| Jiang 2009 [29] | Some concerns | Some concerns | Low risk | Some concerns | High risk |
| Li 2009 [30] | Some concerns | Some concerns | Low risk | Some concerns | High risk |
| Cai 2009 [31] | Some concerns | Some concerns | Low risk | Some concerns | High risk |
| Liao 2009 [32] | Some concerns | Some concerns | Low risk | Some concerns | High risk |
| Cheng 2010 [33] | Some concerns | Some concerns | Low risk | Some concerns | High risk |
| Tian 2010 [34] | Some concerns | Some concerns | Low risk | Some concerns | High risk |
| Wang 2010 [35] | Some concerns | Some concerns | Low risk | Some concerns | High risk |
| Gong 2010 [36] | Some concerns | Some concerns | Low risk | Some concerns | High risk |
| Wang 2010 [37] | Low risk | Some concerns | Low risk | Low risk | High risk |
| Zhang 2010 [38] | Some concerns | Some concerns | Low risk | Some concerns | High risk |
| Xie 2010 [39] | Some concerns | Some concerns | Low risk | Some concerns | High risk |
| Zhang 2011 [40] | Low risk | Some concerns | Low risk | Some concerns | High risk |
| Li 2011 [41] | Some concerns | Some concerns | Low risk | Some concerns | High risk |
| Wu 2011 [42] | Low risk | Some concerns | Low risk | Some concerns | High risk |
| He 2011 [43] | Some concerns | Some concerns | Low risk | Some concerns | High risk |
| Zeng 2011 [44] | High risk | Some concerns | Low risk | Some concerns | High risk |
| Xia 2011 [45] | Some concerns | Some concerns | Low risk | Some concerns | High risk |
| Wu 2012 [46] | Some concerns | Some concerns | Low risk | Some concerns | High risk |
| Tian 2012 [47] | Some concerns | Some concerns | Low risk | Some concerns | High risk |
| Lu 2012 [48] | Some concerns | Some concerns | Low risk | Some concerns | High risk |
| Li 2012 [49] | Some concerns | Some concerns | Low risk | Some concerns | High risk |
| Shao 2012 [50] | Some concerns | Some concerns | Low risk | Some concerns | High risk |
| Zhao 2013 [51] | Low risk | Some concerns | Low risk | Some concerns | High risk |
| Wang 2013 [52] | Some concerns | Some concerns | Low risk | Some concerns | High risk |
| Tang 2013 [53] | Some concerns | Some concerns | Low risk | Some concerns | High risk |
| Zhang 2013 [54] | Some concerns | Some concerns | Low risk | Some concerns | High risk |
| Xu 2013 [55] | Some concerns | Some concerns | Low risk | Some concerns | High risk |
| Song 2014 [56] | Some concerns | Some concerns | Low risk | Some concerns | High risk |
| Zhang 2014 [57] | Some concerns | Some concerns | Low risk | Some concerns | High risk |
| Wang 2014 [58] | Low risk | Some concerns | Low risk | Some concerns | High risk |
| Fang 2014 [59] | Low risk | Some concerns | Low risk | Some concerns | High risk |
| Zhong 2014 [60] | Some concerns | Some concerns | Low risk | Some concerns | High risk |
| Wang 2014 [61] | Some concerns | Some concerns | Low risk | Some concerns | High risk |
| Yao 2014 [62] | Some concerns | Some concerns | Low risk | Some concerns | High risk |
| Yang 2014 [63] | Some concerns | Some concerns | Low risk | Some concerns | High risk |
| Chen 2014 [64] | Low risk | Some concerns | Low risk | Some concerns | High risk |
| Meng 2014 [65] | Some concerns | Some concerns | Low risk | Some concerns | High risk |
| Tang 2014 [66] | Some concerns | Some concerns | Low risk | Some concerns | High risk |
| Fang 2014 [67] | Some concerns | Some concerns | Low risk | Some concerns | High risk |
| Yan 2015 [68] | Some concerns | Some concerns | Low risk | Some concerns | High risk |
| Wei 2015 [69] | Some concerns | Some concerns | Low risk | Some concerns | High risk |
| Cui 2015 [70] | Low risk | Some concerns | Low risk | Some concerns | High risk |
| Zhang 2015 [71] | Low risk | Some concerns | Low risk | Some concerns | High risk |
| Sun 2015 [72] | Some concerns | Some concerns | Low risk | Some concerns | High risk |
| Lu 2015 [73] | Some concerns | Some concerns | Low risk | Some concerns | High risk |
| Zhang 2015 [74] | Low risk | Some concerns | Low risk | Some concerns | High risk |
| Wang 2015 [75] | Low risk | Some concerns | Low risk | Some concerns | High risk |
| Yuan 2015 [76] | Low risk | Some concerns | Low risk | Some concerns | High risk |
| Liao 2015 [77] | Low risk | Some concerns | Low risk | Some concerns | High risk |
| Shi 2015 [78] | Some concerns | Some concerns | Low risk | Some concerns | High risk |
| Li 2015 [79] | Some concerns | Some concerns | Low risk | Some concerns | High risk |
| Ji 2015 [80] | Some concerns | Some concerns | Low risk | Some concerns | High risk |
| Zuo 2015 [81] | Some concerns | Some concerns | Low risk | Some concerns | High risk |
| Zhong 2015 [82] | Some concerns | Some concerns | Low risk | Some concerns | High risk |
| Dong 2015 [83] | Some concerns | Some concerns | Low risk | Some concerns | High risk |
| Wu 2015 [84] | Low risk | Some concerns | Low risk | Some concerns | High risk |
| Jin 2015 [85] | Low risk | Some concerns | Low risk | Some concerns | High risk |
| Li 2015 [86] | Some concerns | Some concerns | Low risk | Some concerns | High risk |
| Li 2015 [87] | Low risk | Some concerns | Low risk | Some concerns | High risk |
| Zhong 2015 [88] | Some concerns | Some concerns | Low risk | Some concerns | High risk |
| Zhang 2015 [89] | Some concerns | Some concerns | Low risk | Some concerns | High risk |
| Han 2015 [90] | Low risk | Some concerns | Low risk | Low risk | High risk |
| Zhu 2015 [91] | Some concerns | Some concerns | Low risk | Some concerns | High risk |
| Chen 2015 [92] | Some concerns | Some concerns | Low risk | Some concerns | High risk |
| Li 2016 [93] | Some concerns | Some concerns | Low risk | Some concerns | High risk |
| Liu 2016 [94] | Some concerns | Some concerns | Low risk | Some concerns | High risk |
| Yang 2016 [95] | Some concerns | Some concerns | Low risk | Some concerns | High risk |
| Yue 2016 [96] | Low risk | Some concerns | Low risk | Some concerns | High risk |
| Yang 2016 [97] | Low risk | Some concerns | Low risk | Some concerns | High risk |
| Mi 2016 [98] | Some concerns | Some concerns | Low risk | Some concerns | High risk |
| He 2016 [99] | Low risk | Some concerns | Low risk | Some concerns | High risk |
| Lai 2016 [100] | Low risk | Some concerns | Low risk | Some concerns | High risk |
| Li 2016 [101] | Low risk | Some concerns | Low risk | Some concerns | High risk |
| Xu 2016 [102] | Some concerns | Some concerns | Low risk | Some concerns | High risk |
| Huang 2016 [103] | Some concerns | Some concerns | Low risk | Some concerns | High risk |
| Zhang 2016 [104] | Some concerns | Some concerns | Low risk | Some concerns | High risk |
| Han 2016 [105] | Some concerns | Some concerns | Low risk | Some concerns | High risk |
| Zhao 2017 [106] | Low risk | Some concerns | Low risk | Some concerns | High risk |
| Wang 2017 [107] | Some concerns | Some concerns | Low risk | Some concerns | High risk |
| Huang 2017 [108] | Low risk | Some concerns | Low risk | Some concerns | High risk |
| Wu 2017 [109] | Some concerns | Some concerns | Low risk | Some concerns | High risk |
| Li 2017 [110] | Some concerns | Some concerns | Low risk | Some concerns | High risk |
| Wang 2017 [111] | Low risk | Some concerns | Low risk | Some concerns | High risk |
| Mei 2017 [112] | Low risk | Some concerns | Low risk | Some concerns | High risk |
| Zhu 2017 [113] | Some concerns | Some concerns | Low risk | Some concerns | High risk |
| Zhu 2017 [114] | Low risk | Some concerns | Low risk | Some concerns | High risk |
| Wu 2017 [115] | Some concerns | Some concerns | Low risk | Some concerns | High risk |
| Shen 2017 [116] | Some concerns | Some concerns | Low risk | Some concerns | High risk |
| Han 2017 [117] | Low risk | Some concerns | Low risk | Some concerns | High risk |
| Jing 2017 [118] | Low risk | Some concerns | Low risk | Some concerns | High risk |
| Wang 2017 [119] | Some concerns | Some concerns | Low risk | Some concerns | High risk |
| Zhou 2017 [120] | Some concerns | Some concerns | Low risk | Some concerns | High risk |
| Gao 2017 [121] | Low risk | Some concerns | Low risk | Some concerns | High risk |
| Lu 2017 [122] | Some concerns | Some concerns | Low risk | Some concerns | High risk |
| Wang 2018 [123] | Some concerns | Some concerns | Low risk | Some concerns | High risk |
| Wang 2018 [124] | Low risk | Some concerns | Low risk | Some concerns | High risk |
| Du 2018 [125] | Low risk | Some concerns | Low risk | Some concerns | High risk |
| Chen 2018 [126] | Some concerns | Some concerns | Low risk | Some concerns | High risk |
| Chen 2018 [127] | Some concerns | Some concerns | Low risk | Some concerns | High risk |
| Wu 2018 [128] | Low risk | Some concerns | Low risk | Some concerns | High risk |
| Huang 2018 [129] | Some concerns | Some concerns | Low risk | Some concerns | High risk |
| Chen 2018 [130] | Low risk | Some concerns | Low risk | Some concerns | High risk |
| Li 2018 [131] | Some concerns | Some concerns | Low risk | Some concerns | High risk |
| Sun 2018 [132] | Low risk | Some concerns | Low risk | Some concerns | High risk |
| Song 2019 [133] | Some concerns | Some concerns | Low risk | Some concerns | High risk |
| Wang 2019 [134] | Low risk | Some concerns | Low risk | Some concerns | High risk |
| Gao 2019 [135] | Low risk | Some concerns | Low risk | Some concerns | High risk |
| Jiang 2019 [136] | Low risk | Some concerns | Low risk | Some concerns | High risk |
| Zou 2019 [137] | Some concerns | Some concerns | Low risk | Some concerns | High risk |
| Zhang 2019 [138] | High risk | Some concerns | Low risk | Some concerns | High risk |
| Pei 2019 [139] | Low risk | Some concerns | Low risk | Some concerns | High risk |
| Zhang 2019 [140] | High risk | Some concerns | Low risk | Some concerns | High risk |
| Shen 2019 [141] | Low risk | Some concerns | Low risk | Some concerns | High risk |
| Su 2019 [142] | Low risk | Some concerns | Low risk | Some concerns | High risk |
| Li 2019 [143] | Low risk | Some concerns | Low risk | Some concerns | High risk |
| Wang 2019 [144] | Low risk | Some concerns | Low risk | Some concerns | High risk |
| Li 2019 [145] | Some concerns | Some concerns | Low risk | Some concerns | High risk |
| Ji 2019 [146] | Low risk | Some concerns | Low risk | Some concerns | High risk |
| Lin 2019 [147] | Some concerns | Some concerns | Low risk | Some concerns | High risk |
| Wang 2019 [148] | Some concerns | Some concerns | Low risk | Some concerns | High risk |
| Xiong 2020 [149] | Some concerns | Some concerns | Low risk | Some concerns | High risk |
| Xian 2020 [150] | Low risk | Some concerns | Low risk | Some concerns | High risk |
| Li 2020 [151] | Low risk | Some concerns | Low risk | Some concerns | High risk |
| Sun 2020 [152] | Some concerns | Some concerns | Low risk | Some concerns | High risk |
| Liu 2020 [153] | Low risk | Some concerns | Low risk | Some concerns | High risk |
| Zhou 2020 [154] | Low risk | Some concerns | Low risk | Some concerns | High risk |
| Tang 2020 [155] | Low risk | Some concerns | Low risk | Some concerns | High risk |
| Tian 2020 [156] | Some concerns | Some concerns | Low risk | Some concerns | High risk |
| Liang 2020 [157] | Low risk | Some concerns | Low risk | Some concerns | High risk |
| Fan 2020 [158] | Some concerns | Some concerns | Low risk | Some concerns | High risk |
| Huang 2020 [159] | Some concerns | Some concerns | Low risk | Some concerns | High risk |
| Zhang 2020 [160] | Low risk | Some concerns | Low risk | Some concerns | High risk |
| Shi 2021 [161] | Some concerns | Some concerns | Low risk | Some concerns | High risk |
| Zhu 2021 [162] | Low risk | Some concerns | Low risk | Some concerns | High risk |
| Chang 2021 [163] | High risk | Some concerns | Low risk | Some concerns | High risk |
| Xie 2021 [164] | Some concerns | Some concerns | Low risk | Some concerns | High risk |
| Wu 2021 [165] | Low risk | Some concerns | Low risk | Some concerns | High risk |
| Ge 2021 [166] | Low risk | Some concerns | Low risk | Some concerns | High risk |
| Li 2022 [167] | High risk | Some concerns | Low risk | Some concerns | High risk |
| Tan 2022 [168] | Low risk | Some concerns | Low risk | Some concerns | High risk |
| Wu 2022 [169] | Low risk | Some concerns | Low risk | Some concerns | High risk |
| Lin 2023 [170] | Low risk | Some concerns | Low risk | Some concerns | High risk |
| Sheng 2023 [171] | Low risk | Some concerns | Low risk | Some concerns | High risk |
| Sun 2023 [172] | Some concerns | Some concerns | Low risk | Some concerns | High risk |

Domains: D1, bias arising from the randomization process; D2, bias due to deviations from intended intervention; D3, bias due to missing outcome data; D4, bias in measurement of the outcome; D5, bias in selection of the reported result.
