# Supplementary material for: Antihypertensive drugs for hyperuricemia in patients with hypertension: a systematic review and network meta-analysis of Chinese trials
Source: BMC Cardiovasc Disord. 2025 Dec 2;25:856. doi: 10.1186/s12872-025-05339-7 (PMC12673777; doi:10.1186/s12872-025-05339-7)
Supplement: Supplementary file 4 — Supplementary Material 4. [file 12872_2025_5339_MOESM4_ESM.docx]

**Supplementary figures legends**


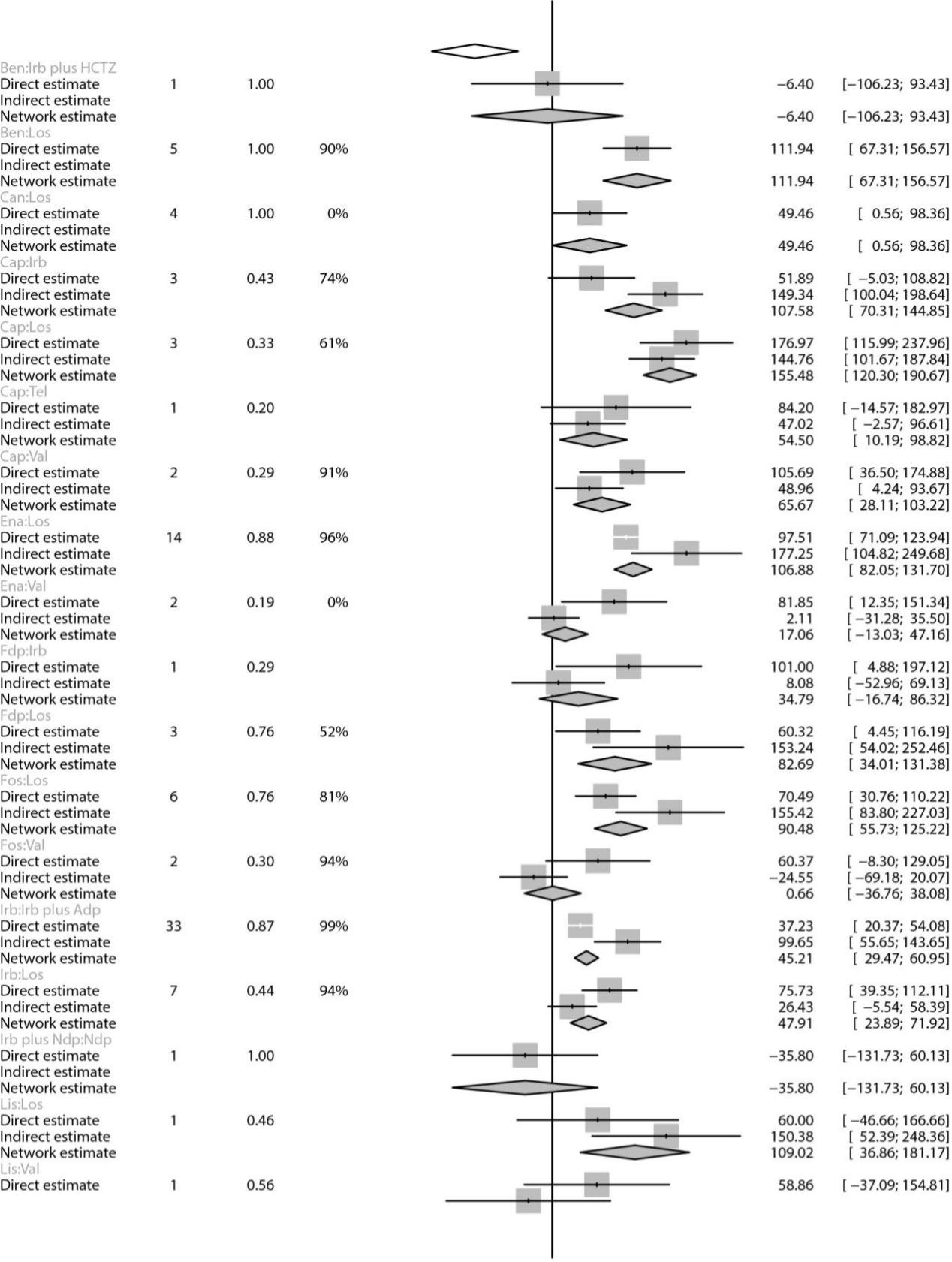


Figure S1. The pair-wise comparisons agents for the change in serum uric acid.


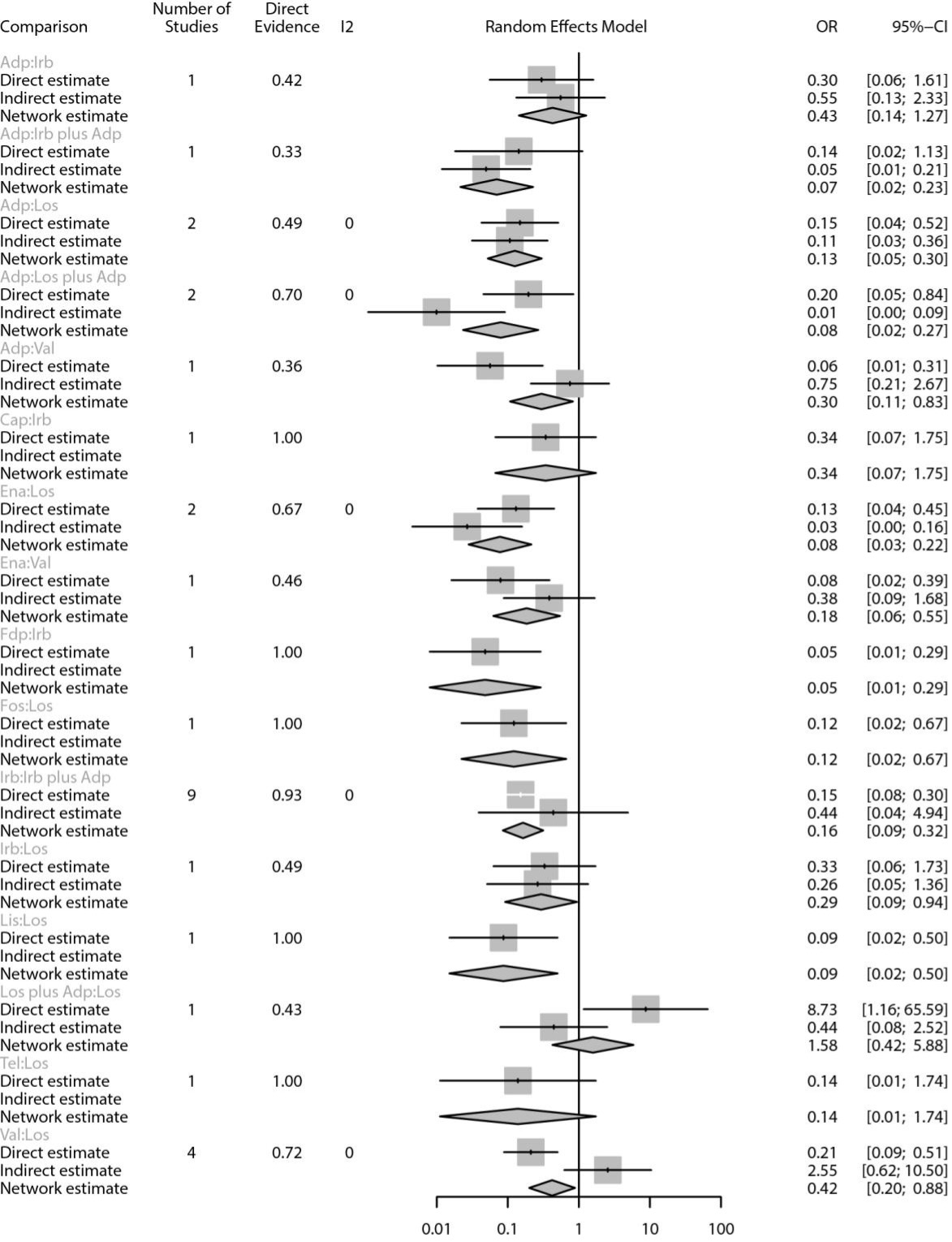


Figure S2. The pair-wise comparisons agents for the incidence of effective rate
